# Affinity proteomics reveals extensive phosphorylation of the Brassica chromosome axis protein ASY1 and a network of associated proteins at prophase I of meiosis

**DOI:** 10.1111/tpj.13752

**Published:** 2017-12-02

**Authors:** Kim Osman, Jianhua Yang, Elisabeth Roitinger, Christophe Lambing, Stefan Heckmann, Elaine Howell, Maria Cuacos, Richard Imre, Gerhard Dürnberger, Karl Mechtler, Susan Armstrong, F. Christopher H. Franklin

**Affiliations:** ^1^ School of Biosciences University of Birmingham Edgbaston Birmingham B15 2TT UK; ^2^ IMP‐IMBA 1030 Vienna Austria; ^3^ Gregor Mendel Institute of Molecular Plant Biology Dr. Bohr‐Gasse 3 1030 Vienna Austria; ^4^ Present address: Faculty of Engineering and Computing Coventry University Coventry CV1 5FB UK; ^5^ Present address: Department of Plant Sciences University of Cambridge Downing Street Cambridge CB2 3EA UK; ^6^ Present address: Leibniz Institute of Plant Genetics and Crop Plant Research (IPK) OT Gatersleben, Corrensstrasse 3 D‐06466 Stadt Seeland Germany

**Keywords:** meiosis, chromosome axis, phosphorylation, LC‐MS/MS, protein–protein interaction, *Brassica oleracea*, *Arabidopsis thaliana*

## Abstract

During meiosis, the formation of crossovers (COs) generates genetic variation and provides physical links that are essential for accurate chromosome segregation. COs occur in the context of a proteinaceous chromosome axis. The transcriptomes and proteomes of anthers and meiocytes comprise several thousand genes and proteins, but because of the level of complexity relatively few have been functionally characterized. Our understanding of the physical and functional interactions between meiotic proteins is also limited. Here we use affinity proteomics to analyse the proteins that are associated with the meiotic chromosome axis protein, ASY1, in *Brassica oleracea* anthers and meiocytes. We show that during prophase I ASY1 and its interacting partner, ASY3, are extensively phosphorylated, and we precisely assign phosphorylation sites. We identify 589 proteins that co‐immunoprecipitate with ASY1. These correspond to 492 Arabidopsis orthologues, over 90% of which form a coherent protein–protein interaction (PPI) network containing known and candidate meiotic proteins, including proteins more usually associated with other cellular processes such as DNA replication and proteolysis. Mutant analysis confirms that affinity proteomics is a viable strategy for revealing previously unknown meiotic proteins, and we show how the PPI network can be used to prioritise candidates for analysis. Finally, we identify another axis‐associated protein with a role in meiotic recombination. Data are available via ProteomeXchange with identifier PXD006042.

## Significance statement

The proteinaceous meiotic chromosome axis is critically important in coordinating meiotic recombination and progression yet due to the level of complexity our understanding of the physical and functional protein interactions involved in this process is limited. Here we immunotarget the Brassica ASY1 protein to analyse the protein network associated with the axis during prophase I, identifying phosphosites in ASY1 and its interacting partner ASY3, revealing a novel axis protein and providing other insights into plant meiosis.

## Introduction

During meiosis, homologous recombination (HR) generates crossovers (COs) that provide genetic variation and promote accurate chromosome segregation at the first meiotic division. The HR pathway has been studied extensively in *Saccharomyces cerevisiae*, and is thought to be broadly similar in plants (Osman *et al*., 2011). HR occurs within the context of profound changes in chromosome organization (Kleckner, 2006). Following replication, sister chromatids are linked by the cohesin complex (Haering and Jessberger, 2012). At the leptotene stage of prophase I, the sister chromatids become organized into linear looped arrays that are conjoined at the loop bases by a proteinaceous axis running along their length. At zygotene, the pairs of homologous chromosomes begin to align and become tightly linked by the synaptonemal complex (SC). This is a highly conserved tripartite structure comprising the chromosome axes with transverse filament (TF) proteins bridging the region between the axes (Page and Hawley, 2004). In many organisms, including plants, mutations leading to defects in axis/SC proteins often have a profound effect on CO formation, whereas in turn recombination pathway mutants can disrupt chromosome morphogenesis (Couteau *et al*., 1999; Grelon *et al*., 2001; Armstrong *et al*., 2002; Li *et al*., 2004; Higgins *et al*., 2005; Ferdous *et al*., 2012).

CO formation is highly coordinated, such that chromosome pairs receive at least one, termed the ‘obligate’ CO (Jones and Franklin, 2006). CO designation is thought to occur early in prophase I, and reduces the probability that another CO will occur in an adjacent region, a phenomenon known as CO interference (reviewed in Berchowitz and Copenhaver, 2010). Precisely how these outcomes are achieved remains to be fully elucidated. Nevertheless, proteins associated with the chromosome axis and SC clearly play an important role (Zickler and Kleckner, 2016). The most extensively studied plant meiotic chromosome axis protein is ASY1 (PAIR2 in rice). Arabidopsis *asy1* mutants fail to synapse and have severely reduced CO formation (Ross *et al*., 1997). In the absence of ASY1, the DMC1 recombinase fails to become stably established on the chromosomes, with the result that interhomologue recombination is severely compromised (Sanchez‐Moran *et al*., 2007). Although not required for axis formation *per se*, ASY1 association with the chromatin is concurrent with axis morphogenesis. Immunolocalization of male meiocytes indicates that it first appears as punctate foci in G2 before progressing to a more linear signal along the entire length of the chromosome axes by leptotene (Armstrong *et al*., 2002). ASY1 remains detectable throughout prophase I, but remodelling of the axis by the AAA+ ATPase, PCH2, during zygotene appears to progressively deplete it from the axis, such that its signal is more obviously associated with the chromatin loops, a process necessary for the normal extension of the SC and the patterned formation of COs (Lambing *et al*., 2015). Taken together, these studies indicate that ASY1 plays important roles in the coordination of axis/SC morphogenesis and recombination to produce the meiosis‐specific bias that favours interhomologue recombination and the maturation of CO‐designated recombination intermediates.

To date, insight into meiosis in plants has largely derived from mutant analysis of individual genes (Mercier *et al*., 2015), identified either through sequence conservation with other species or from mutant or suppressor genetic screens (De Muyt *et al*., 2009; Crismani *et al*., 2012; Girard *et al*., 2014). Global approaches to identify plant meiotic genes and proteins have also been adopted. The transcriptomes of developing anthers undergoing meiosis and of isolated meiocytes have been analysed using microarrays and RNAseq (Chen *et al*., 2010; Tang *et al*., 2010; Aya *et al*., 2011; Deveshwar *et al*., 2011; Libeau *et al*., 2011; Yang *et al*., 2011 and Dukowic‐Schulze *et al*., 2014). These studies reveal a highly complex picture, identifying in the order of 1000–2000 meiotically implicated genes. Moreover, the fact that the relationship between mRNA transcription and the cellular level of the corresponding proteins is nonlinear, the possibility of alternatively spliced meiotic protein variants (Kalsotra and Cooper, 2011; Schmid *et al*., 2013; Sprink and Hartung, 2014 and Wang *et al*., 2014), plus evidence from budding yeast and other species that post‐translational modifications of meiotic proteins play a key role in their function (for example, Rockmill and Roeder, 1991; Lin *et al*., 2010; Attner *et al*., 2013), increases the complexity still further. Proteomic studies present a similarly complex picture (Zhang *et al*., 2017). Analysis of the proteome and phosphoproteome of *Oryza sativa* (rice) anthers identified 4984 proteins and 3203 phosphoproteins associated with early anther development and meiosis (Ye *et al*., 2015), whereas a further study focusing on rice meiocytes identified 1316 proteins (Collado‐Romero *et al*., 2014).

Here we aimed to reduce complexity by using affinity‐based proteomics to immuno‐target the key meiotic axis protein, ASY1, to enrich for associated protein complexes using *Brassica oleracea*, which we have previously shown can be used to provide an enriched source of meiotic tissue for proteomic analysis (Sánchez‐Morán *et al*., 2005; Osman *et al*., 2009). We anticipated that this strategy might also begin to reveal the physical interactions that occur between proteins during prophase I of meiosis. We identify the BoASY1 co‐immunoprecipitating proteins and show that their Arabidopsis counterparts form a coherent protein–protein interaction (PPI) network that, importantly, can be used to prioritize candidates for verification of a meiotic role. We present ICU2, the DNA polymerase α subunit, as proof of principle of this approach. We also describe the discovery of another axis‐associated protein with an apparent role in meiotic recombination and identify multiple phosphorylation sites in BoASY1 and its interacting partner, BoASY3, providing further insights into meiosis in higher plants.

## Results

### Identification of BoASY1 co‐immunoprecipitating proteins

**Figure 1:**
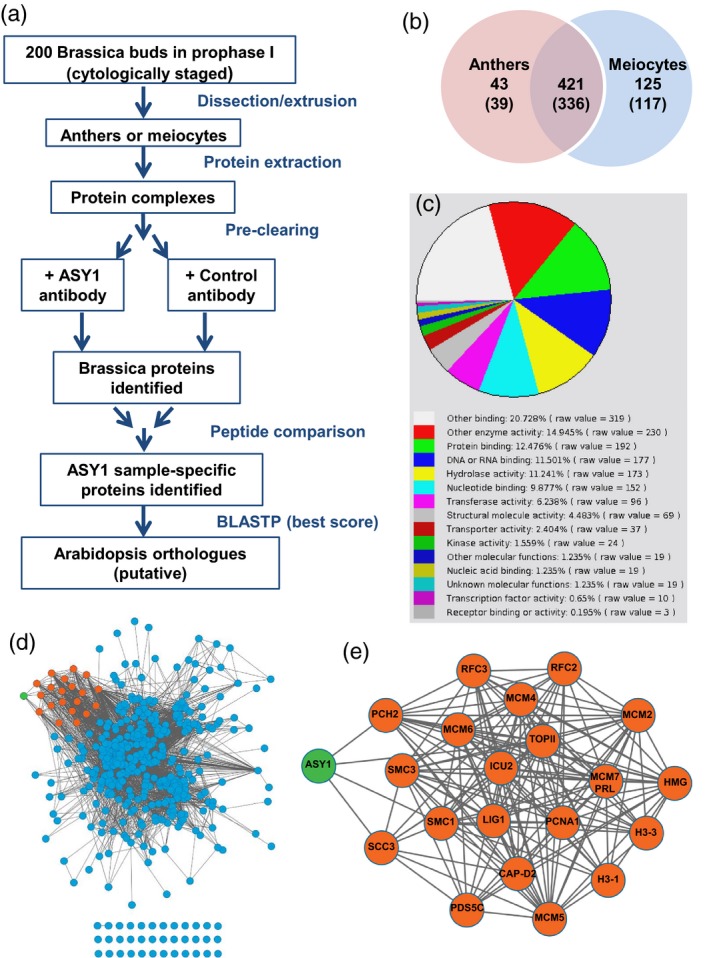
Identification of BoASY1 co‐immunoprecipitating proteins. (a) Summary of workflow for co‐immunoprecipitation (co‐IP) experiments. (b) Numbers of ASY1 sample‐specific proteins identified in Brassica meiotic tissues, with corresponding numbers of putative Arabidopsis orthologues in parentheses. (c) Molecular function of putative Arabidopsis orthologues indicated by gene ontology classification. (d) ASY1 co‐IP network, with nodes representing proteins and edges representing interactions. ASY1 (green) and cluster of cohesin, histone and replication‐related proteins (orange) are highlighted. (e) Detailed view of cluster. Proteins are named according to TAIR.

We carried out co‐immunoprecipitation (co‐IP) of ASY1 from *B. oleracea* to enrich for associated protein complexes (Figure 1a). Anthers (*n* = 200) were used either intact or their contents were extruded to further enrich for meiotic cells (hereafter, these samples are referred to as ‘anthers’ or ‘meiocytes’, respectively). Proteins were extracted under non‐denaturing conditions to preserve meiotic complexes. With the high level of sequence identity between BoASY1 and AtASY1 (83.6%) we could target BoASY1 using an anti‐AtASY1 antibody (Figure S1; Armstrong *et al*., 2002). Parallel control co‐IPs were carried out using non‐specific IgG. Proteins were analysed by in‐solution mass spectrometry (MS) and identified using a combined database comprising *Brassica rapa* sequences (Wang *et al*., 2011) and Brassica sequences obtained from the National Center for Biotechnology Information (NCBI, https://www.ncbi.nlm.nih.gov) in 2010. Putative orthologues in *Arabidopsis thaliana* were identified using the best blastp score. Five anther and four meiocyte data sets were collected. ASY1 was identified in all data sets with 55 peptides in total and up to 75% sequence coverage (Figure S2a), confirming that targeting was successful. In addition, each data set contained several hundred BoASY1 sample‐specific proteins. These ranged from proteins present in all data sets and identified by a relatively large number of unique peptides to those that appeared only once with two unique peptides. To obtain an indication of protein reliability, the raw data from all experiments were searched together and peptide/protein label‐free quantification was carried out using the in‐house tool peakjuggler (unpubl. data; Andersen *et al*., 2017), to determine peak area. Six of the nine data sets were obtained using three technical replicates of each sample, allowing the statistical significance of proteins to be determined using limma analysis (Smyth Gordon, 2004). Proteins showing a fold‐change of ≥5 in sample relative to control and *P *< 0.01 in at least one data set were considered significant in label‐free quantification. In addition, to avoid inadvertently excluding any meiotically relevant proteins, we decided to retain all ASY1 sample‐specific proteins satisfying the minimum identification threshold of two peptides while excluding all proteins identified in any of the control samples. This also allowed us to consider data from the three remaining data sets that lacked replicates. Any proteins that were accepted purely on this qualitative basis were considered less reliable than the quantitatively significant group. Nevertheless, our decision to retain them appeared justified when several were subsequently confirmed as having a meiotic role (see below). Details of both sets of accepted proteins are presented in Table S1.

The group taken forward for further analysis therefore comprised 589 Brassica proteins corresponding to 492 Arabidopsis gene loci. Note that all but one Brassica protein could be assigned a putative Arabidopsis orthologue; the discrepancy between the number of Brassica and Arabidopsis proteins is explained partly by the database containing sequences from several different Brassica species and partly by an ancient *Brassicaceae* lineage‐specific whole‐genome triplication event (Liu *et al*., 2014; Parkin *et al*., 2014), such that in some cases several Brassica proteins indicate the same Arabidopsis orthologue. As expected, there was some overlap between anther and meiocyte data sets, with 421 of the total 589 Brassica proteins (71.5%) identified in both tissue types (336 of 492 for Arabidopsis; Figure 1b); however, 125 Brassica proteins (21.2%) were identified only in meiocytes despite ASY1 being detected equally well in both tissues (117 proteins, 23.8%, for Arabidopsis).

### Chromosome axis and SC‐associated proteins co‐immunoprecipitate with ASY1

Gene ontology (GO) classification of the 492 Arabidopsis orthologues using The Arabidopsis Information Resource website (TAIR, https://www.arabidopsis.org) indicated that the group of ASY1 co‐IP proteins covered a range of molecular functions (Figure 1c). Analysis of GO enrichment relative to the Arabidopsis genome was conducted using panther accessed through the GO consortium website. Further analysis was carried out using the 453 orthologues identified from meiocytes. In both cases, a large number of GO terms were found to be enriched, but notable amongst the Biological Process terms showing the highest fold enrichment were several relating to DNA processing and nucleus organisation (Table S2a), and ‘DNA‐dependent ATP‐ase activity’ was one of the most highly enriched terms for Molecular Function (Table S2b). Several large protein complexes and functional pathways or families were well represented in the ASY1 co‐IP data, so where appropriate we used a combination of the KEGG pathway database (http://www.kegg.jp/kegg/pathway.html) and examination of the relevant literature to group Arabidopsis orthologues accordingly (Table S2).

We identified 12 proteins with a prior confirmed meiotic role in Arabidopsis, including several axis and SC‐associated proteins (Table S2). From the cohesin complex we identified sub‐units SMC1, SMC3 and SCC3 and one of the five Arabidopsis SPO76 cohesin cofactor proteins, PDS5C (Chelysheva *et al*., 2005; Lam *et al*., 2005; Pradillo *et al*., 2015). The SC transverse filament protein, ZYP1a, and the condensin I subunit, CAP‐D2, were also detected (Higgins *et al*., 2005; Smith *et al*., 2014), as were the axis protein, ASY3, and PCH2, an AAA+ ATPase with a role in prophase I axis remodelling, both of which we characterized and published during the course of this study (Ferdous *et al*., 2012; Lambing *et al*., 2015). Given the functional relationship of the HR pathway and the developing axis and SC, it was encouraging that several meiotic recombination proteins immunoprecipitated with ASY1, notably PRD3, required for DNA double‐strand break (DSB) formation (De Muyt *et al*., 2009), and the recombinase DMC1 (Klimyuk and Jones, 1997; Doutriaux *et al*., 1998). Finally, we identified two peptides of the CDK1 homologue, CDKA;1, previously implicated as having a role in meiotic progression (Cromer *et al*., 2012). Most of the previously confirmed meiotic proteins were identified either from both tissue types or solely from meiocyte samples; however, CAP‐D2 was identified only from intact anthers.

Other proteins that have (or are predicted to have) a close association with chromatin were present in the ASY1 co‐IP data (Table S2), including proteins involved in DNA replication and repair, chromatin remodelling proteins, putative transcription factors and regulators, and histone proteins. There were several proteins implicated in the RNA‐dependent DNA methylation (RdDM) pathway, including AGO4 (Table S2). Argonaute proteins have been shown to have important pre‐meiotic and meiotic roles in a range of organisms, including several plant species (Nonomura *et al*., 2007; Olmedo‐Monfil *et al*., 2010; Singh *et al*., 2011; Oliver *et al*., 2014, 2016; Liu and Nonomura, 2016).

Twenty *26S* proteasome and 11 ubiquitination‐related proteins were identified (Table S2), suggesting a close association between these proteins and the meiotic chromosome axis. In animals and yeast, the importance of the ubiquitin–proteasome system (UPS) in regulating key aspects of meiosis, such as recombination and meiotic progression, is well established (Bose *et al*., 2014), and is now also beginning to be elucidated in plants (Wang and Yang, 2006; Zhao *et al*., 2006; He *et al*., 2016).

### ASY1 co‐IP proteins form a coherent protein–protein interaction network

As the ASY1 co‐IP proteins covered a wide range of protein types and GO terms, we carried out network analysis to determine whether they were predicted to interact based on existing data in the public domain. We used an open‐source database of known and predicted protein interactions: STRING (Szklarczyk *et al*., 2015). The STRING network was created using the 492 putative Arabidopsis orthologues of BoASY1 co‐IP proteins (Table S1). The network was visualized using cytoscape (Appendix S1; Figure 1d): 92.7% of proteins (456) formed a single network, with relatively few ‘orphans’ (36), suggesting that they had immunoprecipitated as a coherent group, providing further evidence that the co‐IP approach was successful. It is worth noting that six proteins in the ‘orphans’ group lacked annotation or were otherwise ‘unknown’, and a further five uncharacterized proteins were linked to the main network only by virtue of co‐expression or by being co‐mentioned in public text collections (Appendix S1; Table S2). Given that axis and SC proteins tend to be poorly conserved, these proteins were interesting candidates for further study (see below).

### The ASY1 co‐IP network can be used to prioritize candidates for functional analysis

Candidate proteins were investigated for a meiotic role by cytological examination of chromosome spreads of male meiocytes from homozygous Arabidopsis T‐DNA insertion lines. Initially, we chose candidates based largely on confidence of identification (Table S1) and absence of a previously published role, but we also considered their potential function as inferred from conserved domains, etc. We found several with a strong meiotic mutant phenotype, including ASY3 and PCH2 (Ferdous *et al*., 2012; Lambing *et al*., 2015). In addition, from a sample of 10 candidates exhibiting only a modest or no reduction in fertility, four displayed a relatively minor mutant phenotype, where meiotic defects were clearly observed at the cytological level but were present in only a subset of meiocytes (<10%). Defects included chromosome fragmentation, unresolved interlocks, interbivalent connections, univalency and chromosome bridges at the division stages. Results from the four candidates are summarized in Figures S2 and S4, and Table S4. Interestingly, a phospho‐modified peptide was detected in the N terminus of the Brassica orthologue of one of the candidates (gi257685916; At5g59210), an structural maintenance of chromosomes (SMC) domain protein, and other phosphopeptides were identified in the N‐ and C‐terminal regions of Bra004279 (At1g68060, MAP70‐1) a microtubule‐associated protein (Figure S2b, c). Data from IntAct (Arabidopsis Interactome Mapping Consortium, 2011; Orchard *et al*., 2014), revealed a two‐hybrid array interaction between At5g59210 and At1g68060, thus supporting a direct physical interaction between the *B. oleracea* orthologues of these two proteins in our study.

As mentioned above, many of the ASY1 co‐IP proteins were identified with few unique peptides, appearing in only one or two data sets, and as such might be considered relatively low‐confidence candidates (Table S1). We therefore investigated whether we could use the ASY1 co‐IP STRING network to prioritize candidates for analysis, particularly as the process of identifying and screening homozygous mutants is labour and time intensive. In the network, ASY1 and PCH2 are located in a cluster that contains several histone‐related proteins, cohesin complex components and proteins associated with DNA replication, including RFC complex and MCM family proteins (Figure 1e; Table S2). A member of the MCM family, MCM8, is involved in DMC1‐independent DSB repair in Arabidopsis (Crismani *et al*., 2013), whereas the large subunit of the heteropentameric RFC complex, RFC1, is required for meiotic DSB repair (Liu *et al*., 2013) and interference‐sensitive COs (Wang *et al*., 2012b). Topoisomerase II is necessary for resolving heterochromatic DNA entanglements during female meiosis I in *Drosophila melanogaster* (Hughes and Hawley, 2014), for meiotic chromosome condensation and separation in mice (Li *et al*., 2013), and has a role in mediating CO interference in budding yeast (Zhang *et al*., 2014). Given the clear link between proteins in this cluster and aspects of meiotic DNA metabolism, we decided to investigate other cluster members and, indeed, mutant analysis of several of these, for example ICU2, did suggest a meiotic role.

### ICU2 is required for normal progression through meiosis

**Figure 2:**
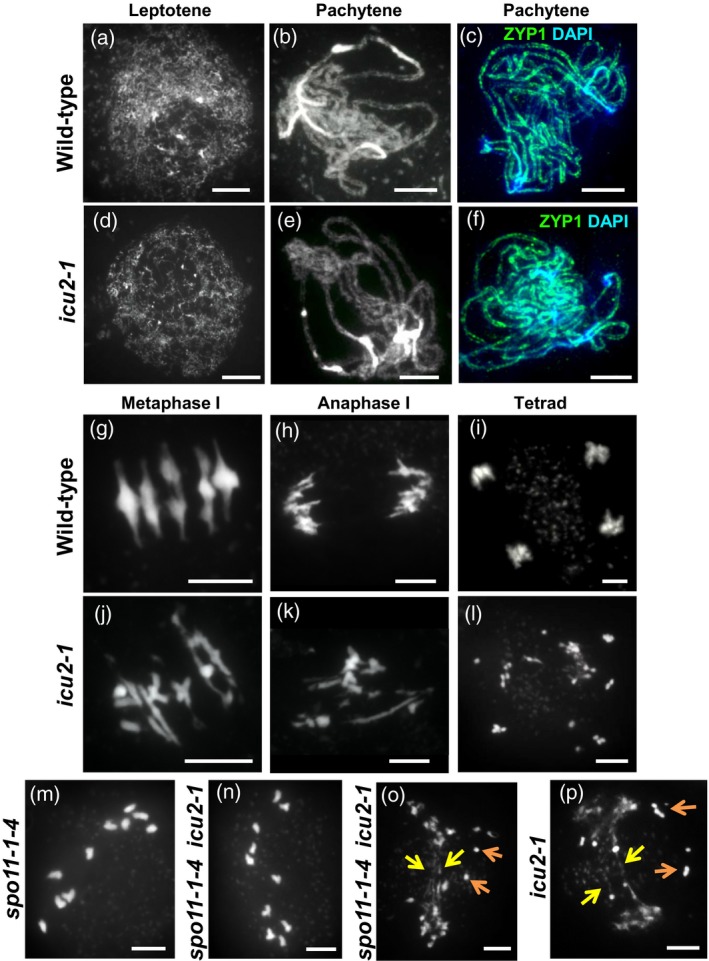
Cytological analysis of *icu2‐1* showing male meiotic chromosome spreads stained with 4′,6‐diamidino‐2‐phenylindole (DAPI). (a–c, g–i) Wild type (WT). (d–f, j–l) *icu2‐1*. (c, f) Immunolocalization of ZYP1 (green), DAPI (blue). (m–p) First division in single and double mutants of *SPO11‐1* and *ICU2*. (m) *spo11‐1‐4* and (n) *spo11‐1‐4 icu2‐1* nucleus with 10 univalents. (o) *spo11‐1‐4 icu2‐1* and (p) *icu2‐1* nucleus with unresolved chromosomal connections (yellow arrows) and fragmentation (orange arrows). Scale bars: 10 μm.

ICU2 (the catalytic subunit of DNA polymerase α) is involved in mediating epigenetic states in Arabidopsis (Barrero *et al*., 2007; Liu *et al*., 2010 and Hyun *et al*., 2013), and has a potential role in HR (Liu *et al*., 2010). To determine whether ICU2 is involved in meiosis we analysed *icu2‐1*, homozygous for a non‐lethal missense allele of the gene (Barrero *et al*., 2007). *icu2‐1* has a pleiotropic phenotype, including early flowering, leaf incurvature, homeotic transformations of some floral parts, reduced plant height and reduced fertility (Barrero *et al*., 2007). In our hands, the fertility of *icu2‐1* was 45.4% (seed count per silique, *n* = 50). Analysis of male meiosis in *icu2‐1* indicated that during most of prophase I the mutant was indistinguishable from wild‐type (WT) Arabidopsis (background En2, 2*n* = 10; Figure 2). Chromosomes appeared as thin threads in leptotene (Figure 2a, d), with homologues becoming fully paired and synapsed by pachytene (Figure 2b, e). ZYP1 immunolocalisation at this stage suggested synapsis was complete (Figure 2c, f). Chromosomes then began to desynapse and condense, and at metaphase I five aligned bivalents were observed in the WT (Figure 2g). Separation of the homologues at anaphase I (Figure 2h) followed by separation of sister chromatids at the second division then resulted in a tetrad of the four haploid products of meiosis (Figure 2i). In *icu2‐1*, however, 44.4% of first divisions appeared aberrant (*n* = 30); nuclei did not exhibit five normal bivalents at metaphase I (Figure 2j), and as homologues began to separate at anaphase I, fragmentation and abnormal chromosomal connections were observed (Figure 2k). This resulted in unbalanced nuclei with fragmented chromosomes at the tetrad stage (Figure 2l). The programmed formation of meiotic DSBs and subsequent recombination and synapsis is dependent on the activity of the SPO11 complex (Bergerat *et al*., 1997). To determine whether the fragmentation/connections observed in *icu2‐1* resulted from unrepaired breaks incurred during pre‐meiotic replication or resulted from defective repair of SPO11‐induced DSBs during recombination, we generated a *spo11‐1‐4 icu2‐1* double mutant. Chromosome spreads of *spo11‐1‐4 icu2‐1* indicated that most meiocytes had a similar phenotype to the *spo11‐1‐4* single mutant, with 10 achiasmate univalents rather than five bivalents at metaphase I (Figure 2m, n). Only 10.0% of nuclei exhibited fragmentation and/or unresolved connections in the double mutant (Figure 2o) compared with 48.3% of nuclei in the *icu2‐1* single mutant (Figure 2p; χ^2^_(1)_ = 19.52, *P* < 0.0001, n = 60). Therefore the *spo11‐1‐4* mutation can largely rescue the phenotype of the *icu2‐1* single mutant, indicating that ICU2 has a role in meiotic recombination.

Analysis of ICU2 therefore provides ‘proof of principle’ of using the co‐IP networks to prioritize particular proteins or key interactions for verification and analysis, and appears to justify our choice of acceptance level in the co‐IP analysis as the protein was identified in the lower confidence qualitative group with only three peptides (see above and Table S1).

We also analysed a homozygous mutant of MCM2, albeit in less detail than *icu2‐1*, and observed mild meiotic defects and a 10% reduction in fertility (Figure S4; Table S4).

### Identification of an axis‐associated protein

As axis and SC proteins tend to be poorly conserved at the primary sequence level, we were interested to note that the ASY1 co‐IP data included several uncharacterized proteins lacking known functional domains (Table S2). As mentioned above, one such protein was subsequently characterized as ASY3 (Ferdous *et al*., 2012). Another protein, encoded by At2g33793, was found to share 23.9% identity and 40.1% similarity with the C‐terminal predicted coiled‐coil region of AtASY3 (Figure S6a). At2g33793 formed links with several other STRING network proteins on the basis of co‐expression, including TOPII, MCM5, CAP‐D2 and PRD3 (Appendix S1). During this project, At2g33793 was independently identified by Mathilde Grelon's group (INRA, France), and was subsequently referred to as *ASY4*.

**Figure 3:**
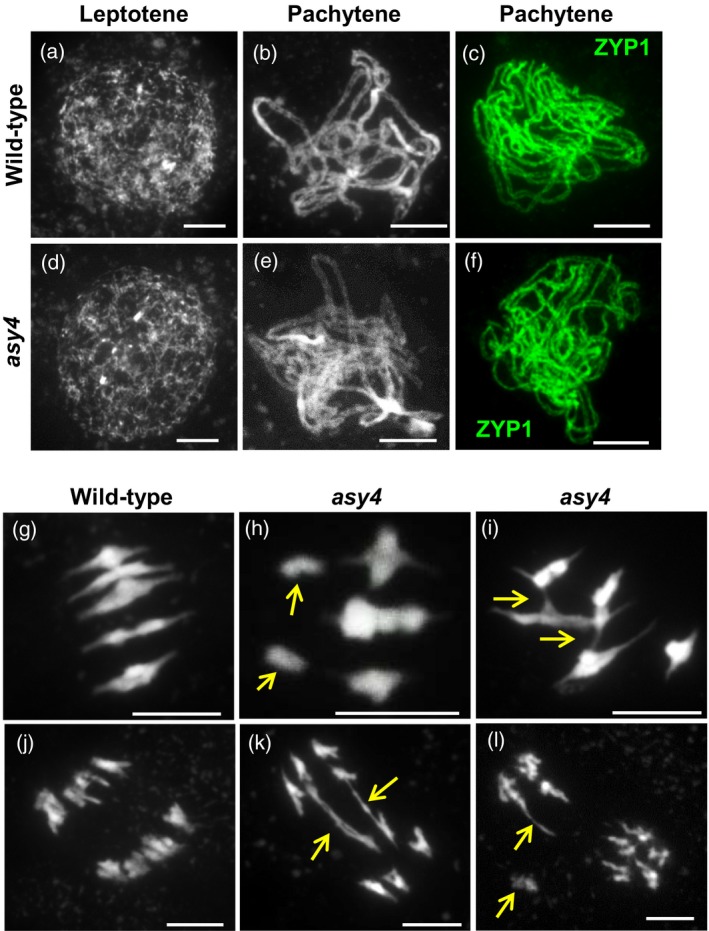
Cytological analysis of *asy4* showing male meiotic chromosome spreads. (a–f) During early meiotic stages *asy4* appears similar to the wild type (WT), with chromosomes becoming paired and synapsed by pachytene. (g) WT metaphase I. (h–i) *asy4* metaphase I with (h) univalents and (i) inter‐bivalent connections. (j) WT anaphase I. (k–l) *asy4* anaphase I, with (k) chromosome bridges and (l) stray chromosome or large fragment and chromosome bridge. DNA is stained with 4′,6‐diamidino‐2‐phenylindole (DAPI). In (c) and (f) the immunolocalization of ZYP1 (green) marks the synaptonemal complex. Arrows indicate relevant features. Scale bars: 10 μm.

Preliminary characterization of a weak mutant allele of ASY4 (Figure S6b, c) indicated normal vegetative growth and silique length, but a slight reduction in fertility based on seed count per silique (mean of 57.50 compared with 60.54 in the WT, *P* < 0.001, *n* = 50). Analysis of meiocytes from *asy4* confirmed a meiotic role (Figure 3). During prophase I, *asy4* appeared similar to the WT, and by pachytene homologues appeared paired and synapsed based on ZYP1 immunolocalization (Figure 3a–f), although we cannot rule out the possibility of short stretches of chromosomes remaining unsynapsed in some nuclei. Most *asy4* nuclei completed meiosis apparently normally; however, at metaphase I, unlike the situation in the WT where five aligned bivalents were invariably observed, a small proportion of *asy4* nuclei contained univalents (2.3%, *n* = 130; Figure 3g, h). Abnormal inter‐bivalent connections were also apparent (Figure 3i). As homologues separated at anaphase I, chromosome bridges were observed in 15.6% (*n* = 32) of nuclei (Figure 3k, l; χ^2^_(1)_ for aberrant nuclei at the first division = 6.28, *P* = 0.012, *n* = 162).

**Figure 4:**
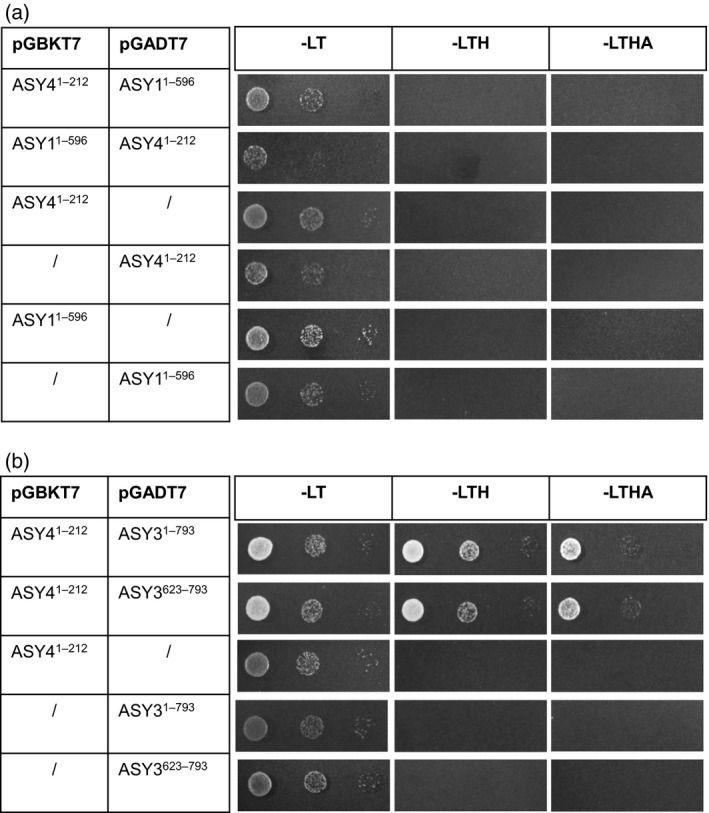
Yeast 2‐hybrid analysis of ASY4. Plasmid constructs were co‐transformed into yeast cells and plated on SD–Leu/–Trp (–LT), SD–Leu/–Trp/–His (–LTH) and SD–Leu/–Trp/–His/–Ade (–LTHA). (a) ASY4 and ASY1: absence of growth on –LTH and –LTHA, but growth on the control medium, –LT, suggested that there was no direct interaction between ASY4 and ASY1. (b) ASY4 and ASY3: growth on –LTH and –LTHA confirmed that the predicted coiled coil‐containing region of ASY3 (amino‐acid residues 623–793) is sufficient for interaction with ASY4.

We then explored the interaction between ASY4 and the other axis proteins, ASY1 and ASY3. Previously we showed that ASY1 and ASY3 directly interact via the C‐terminal predicted coiled‐coil region of ASY3 (Ferdous *et al*., 2012). Given the high sequence similarity between this region of ASY3 and ASY4, we investigated whether ASY4 could also directly interact with ASY1 or, indeed, with ASY3. Yeast two‐hybrid analysis found no direct interaction between ASY1 and ASY4 (Figure 4a). In contrast, in an analysis of full‐length cDNAs from *ASY3* and *ASY4*, yeast growth was enabled even under high‐stringency selection, demonstrating a direct physical interaction between their encoded proteins. Furthermore, as in the interaction between ASY3 and ASY1 (Ferdous *et al*., 2012), the predicted coiled‐coil region of ASY3 (residues 623–793) was sufficient for interaction with ASY4 (Figure 4b). These results confirm that ASY4 is axis‐associated with a potential role in meiotic recombination.

### BoASY1 and BoASY3 are phosphorylated at multiple sites

**Figure 5:**
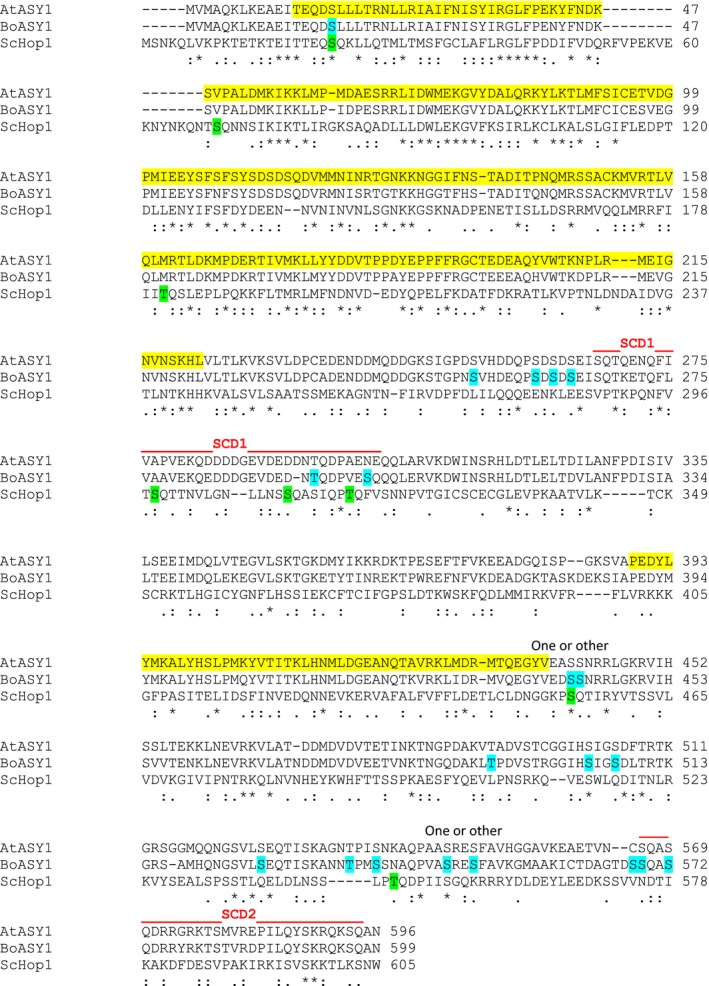
Phosphorylation sites identified in BoASY1. The full‐length sequence of BoASY1 is shown aligned with AtASY1 and its budding yeast orthologue ScHop1 (clustal omega). BoASY1 phospho‐modified residues are highlighted in blue (note that two phospho‐sites could not be precisely determined, as indicated above the sequence). ScHop1 phospho‐sites (Carballo *et al*., 2008) are highlighted in green. S/TQ cluster domains in BoASY1 and AtASY1 are indicated by red lines above the sequence. Predicted HORMA (residues 13‐222) and SWIRM (residues 389‐438) domains are highlighted in yellow in AtASY.

In other organisms the phosphorylation of chromosome axis proteins is important in regulating their activity during meiosis (Rogers *et al*., 2002; Brar *et al*., 2006; Carballo *et al*., 2008; Katis *et al*., 2010; Fukuda *et al*., 2012; Penedos *et al*., 2015; Sakuno and Watanabe, 2015). We were therefore interested in whether MS analysis would enable us to identify phospho‐modified residues in BoASY1 and its interacting partner BoASY3. This proved to be the case. For BoASY1, phospho‐modified forms of 13 different peptides were identified with a total of 18 distinct phospho‐modified Serine (S) or Threonine (T) sites (Table 1). For two of the peptides the precise position of the phosphate group was unclear, but in the majority of cases the position of the phosphorylated residue could be unambiguously determined. Several peptides had doubly phosphorylated forms. Of the 18 phospho‐modified residues, four corresponded to S/TQ motifs, the preferred sites of phosphorylation for the ATM/ATR family of DNA damage response serine/threonine kinases. Notably, all of the phospho‐modified S/TQ sites were located within two S/TQ cluster domains (SCDs), defined as a region where three or more S/TQ motifs occur within a span of up to 100 residues (Traven and Heierhorst, 2005; Figure 5). SCDs are known targets of ATM/ATR. SCD1 is located near the centre of the protein, between HORMA and SWIRM domains, and comprises four S/TQ sites (S267, T272, T294 and S300), with phospho‐modification detected at T294 and S300. AtASY1 also has a SCD in this region, although it differs slightly from the BoASY1 SCD, containing only three S/TQ motifs (S267, T269 and T295) and lacking a site corresponding to S300 in BoASY1 (Figure 5). BoASY1 T294 is conserved, however, corresponding to T295 in AtASY1. Both BoASY1 and AtASY1 also contain a second SCD consisting of three S/TQ motifs close to the C terminus. Two of the S/TQ motifs in this SCD (S569 and S572) lie in close proximity on the same BoASY1 peptide (Figure 5; Table 1), and we identified phospho‐modification at S568, S569 and S572.

**Table 1:** Phosphorylation sites identified in BoASY1 and BoASY3

Protein	Site	Phosphopeptide(s)	Tissue	ptmRS: Best site probabilities
BoASY1	S17	EAEITEQD(S)LLLTR	A	S9(Phospho), 100.00
	S253 and S260	STGPN(S)VHDEQP(S)DSDSEISQTK	M	S6 (Phosho), 97.33; S13 (Phosho), 99.82
	S260	STGPNSVHDEQP(S)DSDSEISQTK	A and M	S13(Phospho), 99.99
	S260 and S262	STGPNSVHDEQP(S)D(S)DSEISQTK		S13(Phospho), 100.00; S15(Phospho), 100.00
	S262	STGPNSVHDEQPSD(S)DSEISQTK		S15(Phospho), 99.85
	S262 and S264	STGPNSVHDEQPSD(S)D(S)EISQTK (with S264)		S15(Phospho), 82.82; S17(Phospho), 90.01
	T294	ETQFLVAAVEKQEDDDGEVDEDN(T)QDPVESQQQLER	A and M	T24(Phospho), 100.00
		QEDDDGEVDEDN(T)QDPVESQQQLER		T13(Phospho), 100.00
	S300	QEDDDGEVDEDNTQDPVE(S)QQQLER	A and M	S19(Phospho), 100.00
		QEDDDGEVDEDN(T)QDPVE(S)QQQLER (with T294)		T13(Phospho), 100.00; S19(Phospho), 100.00
	S442 or S443	MVQEGYVED(S)SNRR or MVQEGYVEDS(S)NRR	A	S10(Phospho), 50.00; S11(Phospho), 50.00
	T493	TNGQDAKL(T)PDVSTR	A and M	T9(Phospho), 100.00
		L(T)PDVSTR		T2(Phospho), 100.00
	S504	GGIH(S)IGSDLTR		S5(Phospho), 98.97
	S504 and S507	GGIH(S)IG(S)DLTR	A and M	S5(Phospho), 100.00; S8(Phospho), 99.88
	S507	GGIHSIG(S)DLTR		S8(Phospho), 100.00
	S526	SAMHQNGSVL(S)EQTISK	M	S11(Phospho), 99.98
	T536	ANN(T)PMSSNAQPVASR	A and M	T4(Phospho), 100.00
	S539	ANNTPM(S)SNAQPVASR	A and M	S7 (Phospho), 99.39
	S547 or S550	ANNTPMSSNAQPVA(S)RESFAVK or	M	S15(Phospho), 50.00; S18(Phospho), 50.00
		ANNTPMSSNAQPVASRE(S)FAVK		
	S568 and S569	ICTDAGTD(S)(S)QASQDRR	A and M	S9 (Phospho), 99.89; S10 (Phospho), 91.24
	S569	ICTDAGTDS(S)QASQDR	A and M	S10(Phospho), 96.10
	S572	ICTDAGTDSSQA(S)QDRR	A and M	S13(Phospho), 98.72
BoASY3	S15	SFGSNFHPS(S)QPR	M	S10(Phospho), 94.53
	S156	GNEMDK(S)PER	A and M	S7(Phospho), 100.00
	S205	A(S)PEYNEDVNSETPEVVK	M	S2(Phospho), 99.67
	T231 or S232	LNQDK(T)SNDDPLTK or LNQDKT(S)NDDPLTK	M	T6(Phospho), 50.00; S7(Phospho), 50.00
	S251 or S253	HHSDTIETD(S)E(S)PEVATR	M	S10(Phospho), 49.72; S12(Phospho), 49.72
	S432 and S441	EK(S)VEPENDFQ(S)PTFGYK	A	S3(Phospho), 100.00; S12(Phospho), 92.49

Phospho‐modified residues are indicated by parentheses. Some peptides were confirmed as doubly phosphorylated. For a few peptides a phospho‐modification could be confirmed, but the precise location within the peptide could not be determined. All sites were identified by ptmRS and manually verified. A (anther) and M (meiocyte) indicate the tissue(s) from which the phosphopeptides were identified.

The remaining phospho‐modified sites in BoASY1 also tend to occur in clusters. Of particular note are S260, S262 and S264, located immediately upstream of SCD1 (Figure 5; Table 1). Phosphorylation at these sites was complex, with both singly and various doubly phosphorylated peptides observed. Interestingly, the multiple acidic residues surrounding the phosphoserines matches the hallmark motif of casein kinase 2 (CK2; Pinna, 2002). Two more loose clusters, each consisting of three phospho‐modified sites within a stretch of up to 15 residues, occur between the SWIRM domain and SCD2. One site in each cluster (T493 and T536) is at a consensus minor CDK1 motif (S/TP) (Figure 5; Table S1).

**Figure 6:**
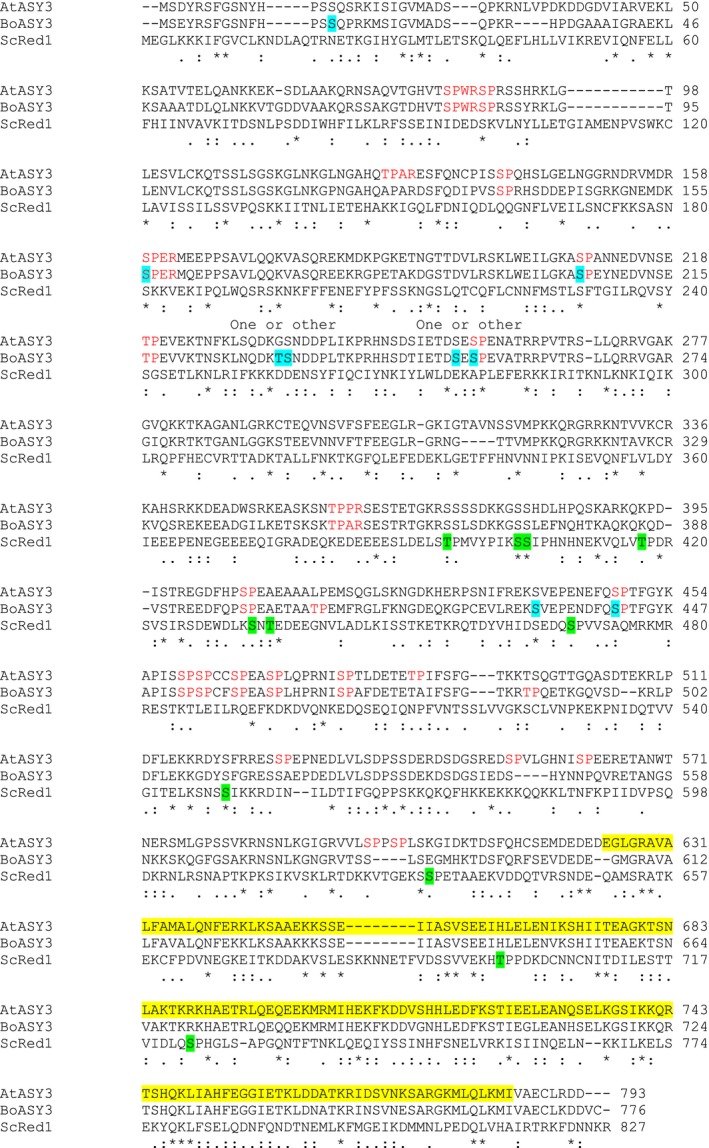
Phosphorylation sites identified in BoASY3. The full‐length sequence of BoASY3 is shown aligned with AtASY3 and ScRed1, the likely functional homologue of ASY3 in yeast (clustal omega). BoASY3 phospho‐modified residues are highlighted in blue. (Note that two BoASY3 phospho‐sites could not be precisely determined, as indicated above the sequence). ScRed1 putative cdc28 sites or cdc28‐independent, but experimentally verified, meiosis‐dependent phospho‐sites (Lai *et al*., 2011) are highlighted in green. Red text indicates minimal (S/T‐P) or full (S/T‐P‐X‐K/R) consensus CDK1 motifs in BoASY3 and AtASY3. A predicted coiled coil region is highlighted in yellow in AtASY3.

In BoASY3 we identified phospho‐modified forms of six different peptides (Table 1). Most carried a single modification, but one was doubly phosphorylated at S432 and S441. The positions of most sites could be unequivocally determined, but in two cases was ambiguous (T231 or S232; S251 or S253). Four of the sites are at consensus CDK1 motifs: S205, S253 and S441 are at minor motifs and S156 is at a full motif (S/TPXK/R; Figure 6). Only one site, S15, is at an S/TQ motif.

## Discussion

In plants, as in other sexually reproducing organisms, the frequency and distribution of COs during meiosis is governed by the functional inter‐relationship between the recombination machinery and the proteinaceous structures that organize the chromosomes during prophase I of meiosis. Thus far our understanding of plant meiosis largely derives from the analysis of around 90 plant meiotic genes, primarily identified through mutant analysis. Although effective, this approach is hampered by several factors. For instance, many of the proteins that are crucial for meiosis are likely to be involved in essential processes in somatic cells. Some genes are duplicated, functionally redundant or, when mutated, produce only subtle phenotypes with little impact on fertility (at least under standard glasshouse growth conditions). Here we have demonstrated that affinity proteomics can be used as an additional approach to identify proteins that play a role in meiosis, and that by targeting a specific component of the meiotic machinery it is possible to begin to define the protein–protein interaction network in which the protein participates.

### Proteins that co‐immunoprecipitate with ASY1 can be organized into a coherent interaction network

Previously, global analyses of the proteomes of developing rice anthers during meiosis and isolated rice meiocytes have identified several thousand proteins, highlighting the complexity of the meiotic proteome (Collado‐Romero *et al*., 2014; Ye *et al*., 2015). Nevertheless, it is likely that the picture is incomplete as Ye *et al*. (2015) identified peptides corresponding to just 10 of at least 28 characterized rice meiotic proteins (Luo *et al*., 2014), and homologues of only 14 and seven Arabidopsis and budding yeast meiotic proteins, respectively.

Adopting a strategy based on affinity purification of meiotic complexes has provided a viable alternative approach, in that focusing on proteins associated with a key meiotic protein, in this case ASY1, substantially reduces complexity and facilitates functional analysis. This approach has enabled us to define a PPI network of 492 nodes that incorporates ASY1. That a substantial proportion of the network proteins are likely to have a meiotic role has been validated using a combination of prior functional knowledge (for example, the presence of CAP‐D2, ZYP1 and PRD3; Higgins *et al*., 2005; De Muyt *et al*., 2009; Smith *et al*., 2014) and functional analysis (this study; Ferdous *et al*., 2012; Lambing *et al*., 2015). It is apparent from this analysis that although some network proteins such as ASY3, PCH2, ICU2 and PRD3 have major meiotic roles, this is not the case for a significant proportion. Mutant analysis of a small sample suggests that many of the network proteins may have only a minor effect on meiosis; however, this is based on a preliminary analysis, and hence we cannot rule out that the mild mutant phenotypes are the result of functional redundancy or the fact that the analyses were conducted only under standard growth conditions. It is important to note that given their subtle mutant phenotypes, a meiotic role for these proteins is unlikely to have been detected using previous approaches, such as fertility screening.

A further important point is that the presence of a protein within the PPI network does not necessarily imply a direct molecular interaction with ASY1 or indeed any other component. Additional analyses are required to determine such interactions. For example, Y2H analysis confirmed a direct interaction between ASY1 and ASY3 (Ferdous *et al*., 2012), whereas this is not the case for ASY1 and ZYP1. Indeed, installation of ZYP1 to form the SC is dependent on PCH2‐mediated depletion of ASY1 from the axis (Lambing *et al*., 2015).

Using the network led us to prioritize ICU2, the catalytic subunit of Arabidopsis DNA polymerase α, for analysis, and we confirmed that it has a role in meiotic recombination. RFC1 was proposed to be involved in DNA lagging‐strand synthesis during double Holliday junction formation (Wang *et al*., 2012a), and DNA leading‐strand synthesis was found to be important for the formation of interference‐sensitive COs in Arabidopsis (Huang *et al*., 2015). The precise role of ICU2 will require further study.

Cytological analysis suggested a potentially interesting mutant phenotype for At5g59210, the protein product of which is predicted to contain extensive coiled‐coil regions. Further work will be required to fully characterize the role of this protein, but the identification of a phospho‐site near the N‐terminus of its Brassica orthologue is interesting, particularly as IntAct indicates an interaction with MAP70‐1, a plant‐specific microtubule‐associated protein, the Brassica orthologue of which was also found to be phosphorylated. We have not yet investigated MAP70‐1 for a meiotic role. Any analysis would need to address the fact that it is part of a multigene family, sharing a high degree of identity with three other proteins (Korolev *et al*., 2005), and that the Brassica orthologues of all four proteins were present in the ASY1 co‐IP data (Tables S1 and S2). Consistent with the identification of microtubule‐associated proteins, several β‐tubulin and α‐tubulin proteins were also present in the co‐IP data.

Of the 492 Arabidopsis loci submitted for STRING analysis, 11 were unknown/uncharacterized and six could not be incorporated into the PPI network. These proteins were thought to be good candidates for investigation for a meiotic role, which led to the identification and preliminary characterization of ASY4. Although we were able to analyse only a weak mutant allele of ASY4, its cytological phenotype of impaired CO formation, together with its high degree of similarity to the C terminal of ASY3, and the confirmation of a direct Y2H interaction between the two proteins, strongly supports an axis‐associated role for the protein. No direct Y2H interaction with ASY1 was detected, suggesting that ASY4 may have been co‐immunoprecipitated by an indirect interaction with ASY1 via ASY3, thus illustrating a further advantage of using an affinity proteomics approach in that by targeting meiotic complexes, secondary and even higher order protein interactors might be identified. An indirect interaction with ASY1 might explain why we identified relatively few unique peptides of ASY4 compared with ASY1 and ASY3 (3, 55 and 39, respectively), although this could also have been influenced by its smaller size (ASY4 has a predicted molecular weight of 24.69 kDa compared with 67.21 kDa for ASY1 and 88.00 kDa for ASY3).

### Protein phosphorylation

Mass spectrometry (MS) revealed that *in vivo* BoASY1 is extensively phosphorylated at prophase I of meiosis. Amongst the 18 identified sites there were four S/TQ motifs distributed between two SCDs. SCD1 is located near the centre of the protein, between the HORMA and SWIRM domains, and SCD2 is near the C terminus. Sequence alignment suggests a comparable arrangement in AtASY1, although there appears to be an additional S/TQ motif in SCD1 of BoASY1 (Figure 5). Although further studies will be required to determine whether phosphorylation of the S/TQ residues in SCD1 and SCD2 is of functional significance, comparison with Hop1 in budding yeast (Figure 5) suggests that this is possible, at least for some of the S/TQ sites in SCD1 (Carballo *et al*., 2008). Hop1 contains eight S/TQ motifs; three of them form an SCD located just downstream of the HORMA domain, and all three are phosphorylated *in vivo* during meiosis. Phosphorylation at T318 has the greatest effect in promoting Hop1‐dependent interhomologue recombination (Carballo *et al*., 2008), and phosphorylation at S298 promotes stable interaction of HOP1 and Mek1 effector kinase on the chromosomes following initial phospho‐T318 mediated Mek1 recruitment (Penedos *et al*., 2015). Similar to Hop1, SCD1 is found near the centre of the protein between the HORMA and SWIRM domains in BoASY1 and AtASY1. A full‐length alignment of BoASY1 with Hop1 (Figure 5) suggests that residue T294 in SCD1 corresponds in position to Hop1 T318. As is the case for T318 in Hop1, a flanking S/TQ motif (S300) is also phosphorylated. Although it is reasonable to speculate that the S/TQ sites within SCD1 and the Hop1 SCD may be functionally comparable, this is clearly not the case for SCD2, which is absent from the budding yeast protein. SCD2 is conserved in the rice HORMA domain protein, OsPAIR2, however, and has also been reported to undergo phosphorylation (Ye *et al*., 2015). This occurred at S579, which corresponds to S572 in BoASY1. A second OsPAIR2 phospho‐site was detected nearby at S569, a non‐S/TQ site. Nevertheless, the significance of phosphorylation in the ASY1 C terminal remains obscure, as it appears that this SCD is present in the orthologues of some plant species, but not in others.

The significance of the tendency for the non‐S/TQ phosphosites in BoASY1 to also occur in clusters remains to be determined. The cluster S260, S262 and S264 just upstream of SCD1 (Figure 5; Table 1) is particularly interesting because the multiple acidic residues surrounding the phospho‐serines matches the hallmark motif of casein kinase II (Pinna, 2002). CK2 motifs were recently identified amongst irradiation and ATM/ATR‐dependent upregulated phosphorylation sites in Arabidopsis, although it remains to be seen whether these sites are actually targeted by CK2 in an ATM/ATR‐dependent manner (Roitinger *et al*., 2015). Phosphorylation at minor CDK1 sites within the two clusters situated between the SWIRM domain and SCD2 may also be of significance, particularly as CDKA;1 was amongst the proteins we identified, albeit with only two peptides.

The most striking feature of the BoASY3 phospho‐sites is that all seven are located in the N‐terminal region of the protein (Figure 6). This is consistent with an earlier functional analysis of ASY3 that showed that the C‐terminal coiled‐coil region of the protein (residues 623–793) is involved in its interaction with ASY1 in Arabidopsis (Ferdous *et al*., 2012), and may therefore be inaccessible for signalling. Four of the sites were at consensus CDK1 motifs: one at a full motif and three at minor motifs. A single phospho‐site, at position S81, was identified in the rice ASY3 orthologue, PAIR3, and was also at a minor CDK1 motif (Ye *et al*., 2015). Red1, the budding yeast orthologue of BoASY3, contains seven putative target sites of Cdc28 (CDK1) and at least four Cdc28‐independent phosphorylation sites (Lai *et al*., 2011); however, in a full‐length alignment of BoASY3 and Red1, only S432 and S441 lie in close proximity to a Red1 phosphosite (S469; Figure 6). Of these, S441 is a minimal S/TP motif, like Red1 S469. Functional analysis of phosphorylation in Red1 suggested that it was non‐essential for its functions in meiosis (Lai *et al*., 2011), so it will be interesting to investigate any potential role for ASY3 and ASY1 phosphorylation in future studies.

Besides CDKA;1, several other kinases and phosphatases were identified in the ASY1 co‐IP data, including the protein phosphatase 2A subunits PP2AA2 and PP2A‐3 (Tables S1 and S2). PP2A has been implicated in a number of meiotic roles in animals and yeast (e.g. Lu *et al*., 2002; Kitajima *et al*., 2006; Riedel *et al*., 2006; Nolt *et al*., 2011; Tang *et al*., 2016), and is regulated by the UPS during mouse oocyte maturation (Yu *et al*., 2015). It remains to be established whether it, or any of the kinases identified in our study, has a role in plant meiosis, however.

### Proteins associated with other cellular processes

The ASY1 co‐IP data contained multiple components of several large complexes and functional pathways, such as the *26S* proteasome, the ubiquitination system and the spliceosome (Table S2). Because of their participation in a wide range of cellular functions, one could argue that these proteins were recovered simply as a result of non‐specific protein interactions. It is therefore important to emphasize that they were identified either as significant in label‐free quantification or as ASY1 sample‐specific (absent from all control data sets), suggesting that at least some of the complex/pathway components were isolated on the basis of a specific interaction with the ASY1 meiotic complex. Indeed, an examination of the literature provides several indications of a close association between these particular protein complexes/pathways and meiotic chromatin. For example, HEI10, a mammalian RING domain protein with E3 ubiquitin‐ligase activity (Ward *et al*., 2007), and related proteins in budding yeast (Zip3), *Sordaria macrospora*, Arabidopsis and rice (HEI10) are required for CO formation, and have been shown to localize to discrete foci along meiotic chromosomes (Agarwal and Roeder, 2000; Chelysheva *et al*., 2012; Wang *et al*., 2012a; De Muyt *et al*., 2014; Qiao *et al*., 2014). Furthermore, studies indicate that Zip3 in yeast and RNF212 (its mammalian orthologue) and HEI10 in mouse mediate the recruitment of proteasomes to chromosome axes to regulate axis morphogenesis, homologue pairing, synapsis and meiotic recombination (Ahuja *et al*., 2017; Rao *et al*., 2017). Examination of the *Caenorhabditis elegans* germline provides further evidence that proteasome recruitment to the chromosome axes is an evolutionarily conserved feature of meiosis (Ahuja *et al*., 2017).

Around 400 splicing‐related proteins have been predicted or confirmed in Arabidopsis (Wang and Brendel, 2004; Koncz *et al.,* 2012). We identified 25 spliceosome‐related proteins (Table S2), including PRL1 from the spliceosome‐activating NineTeen Complex (NTC) core and three NTC‐associated proteins, as defined by Monaghan *et al.,* (2009). There is increasing evidence of a role for the NTC in the coordination of DNA damage responses (reviewed in Koncz *et al.,* 2012). Interestingly, Ye *et al*. (2015) found that the RNA splicing pathway was extensively phosphorylated in rice anthers at around the time of meiosis (as indeed were the DNA synthesis and RdDM pathways, which are also well represented in our data). Although any association of spliceosome factors with meiotic chromosomes or a DNA repair role in meiosis remains to be established, our analysis may be a pointer in this direction.

### Technical considerations

Although we anticipated that it might be feasible to use anthers to identify proteins that co‐IP with ASY1, we were concerned that meiocytes represent only a small proportion of the tissue, potentially compromising our ability to identify less abundant meiotic proteins. Our data reveal that although several of the proteins with a prior confirmed role in meiosis could be identified from anthers, most were identified exclusively from the meiocyte‐enriched samples. This was also the case for ICU2 and ASY4. Other experiments carried out in our lab suggest that certain meiotic proteins, notably ZYP1, are more easily recovered from intact anthers than extruded meiocytes, however, and hence there may be technical reasons favouring their detection from this tissue. In this context, it is interesting that during native meiotic protein extraction in budding yeast, the ZYP1 orthologue Zip1 appears to be far less stable than ASY1 and ASY3 homologues Hop1 and Red1 (Lin *et al.,* 2010). Thus, although it is clear that the additional effort in preparing meiocyte‐enriched material was justified, it seems that analysis of both tissue types provides the most comprehensive picture.

The decision to retain the lower confidence group of all ASY1 sample‐specific proteins satisfying the minimum identification threshold of two peptides appeared justified for two reasons. First, several of them were found to have a meiotic role, and second, over 90% of the identified proteins formed a single PPI network; however, we cannot rule out that in some cases low‐confidence proteins may have appeared to be sample specific by chance. There are several biological reasons why genuine interactors might appear with low confidence. Low‐abundance proteins and proteins that form only transient interactions with the ASY1 complex may be under‐represented in samples (extreme examples being protein kinases and phosphatases). Indirect interactors or proteins that form only weak interactions with the complex may also be more difficult to detect. Further sample enrichment to target a substage of prophase I may begin to address some of these limitations, as would targeting other proteins in the ASY1 interaction network in order to confirm and extend the network, enabling a comprehensive picture of meiotic interactions to emerge. This, together with ongoing technical improvements in sample preparation methods and MS analysis, should help to increase our ability to identify genuine protein interactors that currently lie at the borderline of detection.

## Experimental procedures

### Plant material, nucleic acid extraction and mutation site mapping

*Arabidopsis thaliana* ecotypes Columbia (0) and Enkheim‐2 (En‐2) and *B. oleracea* var. alboglabra A12DHd were used for WT analysis. Arabidopsis seed stocks were obtained from the Nottingham Arabidopsis Stock Centre (http://arabidopsis.info). Plants were grown, Arabidopsis material was harvested and nucleic acid extractions were carried out as previously described (Higgins *et al.,* 2004). T‐DNA insertion sites of mutant lines were confirmed by PCR and, in the case of *asy4,* by sequencing. The missense mutation of *icu2‐1* was confirmed by sequencing and tetra‐primer ARMS‐PCR (Ye *et al.,* 2001). Primer details are listed in Appendix S2.

### Co‐immunoprecipitation analysis

Brassica meiotic tissue was collected as previously described (Sánchez‐Morán *et al*., 2005). Co‐IP analysis was based on a previously described method, with minor modifications (Osman *et al*., 2013). Full details of the procedure are available in Appendix S2.

### Bioinformatic analysis

Brassica proteins were used to identify putative *A. thaliana* orthologues using best blastp 2.6.0 score (with an acceptance threshold of an E‐value of 1*e* ^−5^) against TAIR 10 protein sequences (https://www.arabidopsis.org). GO categorization of *A. thaliana* orthologues was carried out using the TAIR website (https://www.arabidopsis.org/tools/bulk/go/index.jsp). GO enrichment analysis was carried out using panther accessed through the GO consortium website (http://geneontology.org). The KEGG pathway database was used to predict functional pathways for Arabidopsis orthologues (http://www.kegg.jp/kegg/pathway.html; Kanehisa *et al*., 2016). PPI networks of Arabidopsis orthologues were generated using STRING 10.5 (http://string-db.org; Szklarczyk *et al*., 2015), using default settings. The resulting network and protein description files were used to produce the networks in cytoscape 3.5.1 (http://www.cytoscape.org). Sequence alignments were carried out using clustal omega (Sievers *et al*., 2011) or emboss needle (Rice *et al*., 2000), accessed through the EMBL‐EBI website (https://www.ebi.ac.uk).

### Antibody production

The AtZYP1B C‐terminal antibody was produced using a previously described procedure (Ferdous *et al*., 2012) with primers ZYP1B‐C‐F and ZYP1B‐C‐R (Appendix S2). Polyclonal antiserum against the recombinant protein was raised in rabbit (Orygen Antibodies Ltd.; http://www.orygen.co.uk).

### Cytological procedures

Cytological procedures were carried out as previously described (Higgins *et al.,* 2004). Antibodies were used as follows: anti‐AtASY1 (rat, 1/1000 dilution) and anti‐AtZYP1B‐C (rabbit, 1/500 dilution). DNA was stained with 1 μg ml^−1^ 4′,6‐diamidino‐2‐phenylindole (DAPI) in Vectashield.

### Yeast 2‐hybrid analysis

Yeast 2‐hybrid analysis was carried out as previously described (Ferdous *et al*., 2012). Details of primers used for plasmid construction are presented in Appendix S2.

### Statistical procedures

Fertility in WT and mutant plants was compared using single‐factor anova. Chi‐square (χ^2^) tests were carried out using graphpad prism 7 (https://graphpad.com) using Yate's correction.

## Accession numbers

The MS proteomics data have been deposited to the ProteomeXchange Consortium via the PRIDE partner repository (Vizcaíno *et al*., 2016), with the identifier PXD006042. The following lines were used for mutant analysis: At5g46070, SALK_016366; At3g52140, SALK_046271; At5g42220, SALK_151742; At5g59210, GABI_094G05; *mcm2* (At1g44900), SALK_023429; *icu2‐1* (At5g67100), N329; *spo11‐1‐4* (At3g13170), WiscDsLox461‐464J19 (Roberts 2010); *asy4* (At2g33793), SAIL_886_D04.

## Conflicts of interest

The authors declare no known conflicts of interest.

## Supporting information


**Figure S1.** Targeting BoASY1 using an anti‐AtASY1 antibody.

**Figure S2.** Protein sequence coverage.

**Figure S3.** Mutant analysis of three meiotic candidates.

**Figure S4.** Mutant analysis of At5g59210.

**Figure S5.** Mutant analysis of meiotic candidate MCM2.

**Figure S6.** Alignment of ASY4 (At2g33793) with ASY3 and mapping of T‐DNA insertion SAIL_886_D04 in *asy4*.

**Table S1.** ASY1 sample‐specific Brassica proteins with their putative Arabidopsis orthologues.

**Table S2.** Gene ontology enrichment analysis of ASY1 sample‐specific proteins.

**Table S3.** Functional grouping of ASY1 sample‐specific proteins.

**Table S4.** Summary of analysis of meiotic candidates.

**Appendix S1.** PPI network of ASY1 sample‐specific proteins in Cytoscape format.

**Appendix S2.** Supporting experimental procedures.
