# Supplementary material for: Affinity proteomics reveals extensive phosphorylation of the Brassica chromosome axis protein ASY1 and a network of associated proteins at prophase I of meiosis
Source: Plant J. 2017 Dec 2;93(1):17–33. doi: 10.1111/tpj.13752 (PMC5767750; doi:10.1111/tpj.13752)
Supplement: Supplementary file 1 — Figure S1. Targeting BoASY1 using an anti‐AtASY1 antibody. [file TPJ-93-17-s001.pdf]

a

```
AtASY1               1 MVMAQKLKEAEITEQDSLLLTRNLLRIAIFNISYIRGLFPEKYFNDKSVP     50
                       |||||||||||||||||||||||||||||||||||||||||.||||||||
BoASY1               1 MVMAQKLKEAEITEQDSLLLTRNLLRIAIFNISYIRGLFPENYFNDKSVP     50

AtASY1              51 ALDMKIKKLMPMDAESRRLIDWMEKGVYDALQRKYLKTLMFSICETVDGP    100
                       |||||||||:|:|.||||||||||||||||||:||||||||.|||:|:||
BoASY1              51 ALDMKIKKLLPIDPESRRLIDWMEKGVYDALQKKYLKTLMFCICESVEGP    100

AtASY1             101 MIEEYSFSFSYSDSDSQDVMMNINRTGNKKNGGIFNSTADITPNQMRSSA    150
                       |||||||:|||||||||||.|||:|||.||:||.|:||||||.|||||||
BoASY1             101 MIEEYSFNFSYSDSDSQDVRMNISRTGTKKHGGTFHSTADITQNQMRSSA    150

AtASY1             151 CKMVRTLVQLMRTLDKMPDERTIVMKLLYYDDVTPPDYEPPFFRGCTEDE    200
                       |||||||||||||||||||:|||||||:||||||||.|||||||||||:|
BoASY1             151 CKMVRTLVQLMRTLDKMPDKRTIVMKLMYYDDVTPPAYEPPFFRGCTEEE    200

AtASY1             201 AQYVWTKNPLRMEIGNVNSKHLVLTLKVKSVLDPCEDENDDMQDDGKSIG    250
                       ||:||||:|||||:|||||||||||||||||||||.||||||||||||.|
BoASY1             201 AQHVWTKDPLRMEVGNVNSKHLVLTLKVKSVLDPCADENDDMQDDGKSTG    250

AtASY1             251 PDSVHDDQPSDSDSEISQTQENQFIVAPVEKQDDDDGEVDEDDNTQDPAE    300
                       |:||||:||||||||||||:|.||:||.||||:|||||||| ||||||.|
BoASY1             251 PNSVHDEQPSDSDSEISQTKETQFLVAAVEKQEDDDGEVDE-DNTQDPVE    299

AtASY1             301 NEQQLARVKDWINSRHLDTLELTDILANFPDISIVLSEEIMDQLVTEGVL    350
                       ::|||.||||||||||||||||||:|||||||||.|:|||||||..||||
BoASY1             300 SQQQLERVKDWINSRHLDTLELTDVLANFPDISIALTEEIMDQLEKEGVL    349

AtASY1             351 SKTGKDMYIKKRDKTPESEFTFVKEEADGQIS--PGKSVAPEDYLYMKAL    398
                       |||||:.|...|:|||..||.|||:||||:.:  ..||:|||||:|||||
BoASY1             350 SKTGKETYTINREKTPWREFNFVKDEADGKTASKDEKSIAPEDYMYMKAL    399

AtASY1             399 YHSLPMKYVTITKLHNMLDGEANQTAVRKLMDRMTQEGYVEASSNRRLGK    448
                       ||||||:||||||||||||||||||.||||:|||.||||||.||||||||
BoASY1             400 YHSLPMQYVTITKLHNMLDGEANQTKVRKLIDRMVQEGYVEDSSNRRLGK    449

AtASY1             449 RVIHSSLTEKKLNEVRKVLAT-DDMDVDVTETINKTNGPDAKVTADVSTC    497
                       |||||.:||.||||||||||| |||||||.||:|||||.|||:|.||||.
BoASY1             450 RVIHSVVTENKLNEVRKVLATNDDMDVDVEETVNKTNGQDAKLTPDVSTR    499

AtASY1             498 GGIHSIGSDFTRTKGRSGGMQQNGSVLSEQTISKAGNTPISNKAQPAASR    547
                       |||||||||.||||||| .|.||||||||||||||.|||:|:.|||.|||
BoASY1             500 GGIHSIGSDLTRTKGRS-AMHQNGSVLSEQTISKANNTPMSSNAQPVASR    548

AtASY1             548 ESFAVHGGAVK---EAETVNCSQASQDRRGRKTSMVREPILQYSKRQKSQ    594
                       |||||.|.|.|   :|.| :.||||||||.||||.||:||||||||||||
BoASY1             549 ESFAVKGMAAKICTDAGT-DSSQASQDRRYRKTSTVRDPILQYSKRQKSQ    597

AtASY1             595 AN    596
                       ||
BoASY1             598 AN    599
```

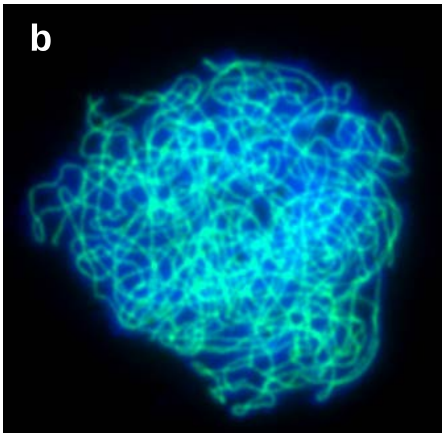

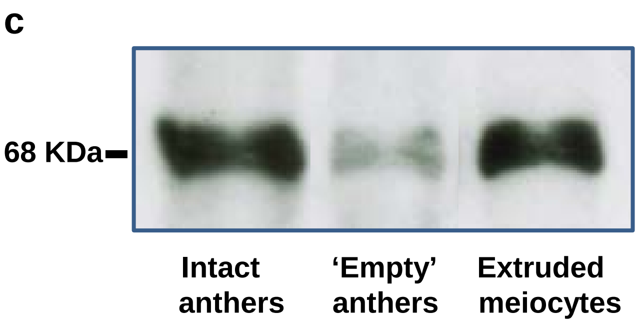

Figure S1. Targeting BoASY1 using an anti-AtASY1 antibody.
(a) Full-length alignment of AtASY1 and BoASY1 using EMBOSS Needle (www.ebi.ac.uk). (**b**) Immunolocalisation of ASY1 (green) at leptotene in *B. oleracea* meiocyte. DNA is stained with DAPI (blue). **(c)** Immunoblot of ASY1 in *B. oleracea* meiotic tissues (loading is equivalent to 10 anthers per lane).
