# Supplementary material for: Affinity proteomics reveals extensive phosphorylation of the Brassica chromosome axis protein ASY1 and a network of associated proteins at prophase I of meiosis
Source: Plant J. 2017 Dec 2;93(1):17–33. doi: 10.1111/tpj.13752 (PMC5767750; doi:10.1111/tpj.13752)
Supplement: Supplementary file 2 — Figure S2. Protein sequence coverage. [file TPJ-93-17-s002.pdf]

a

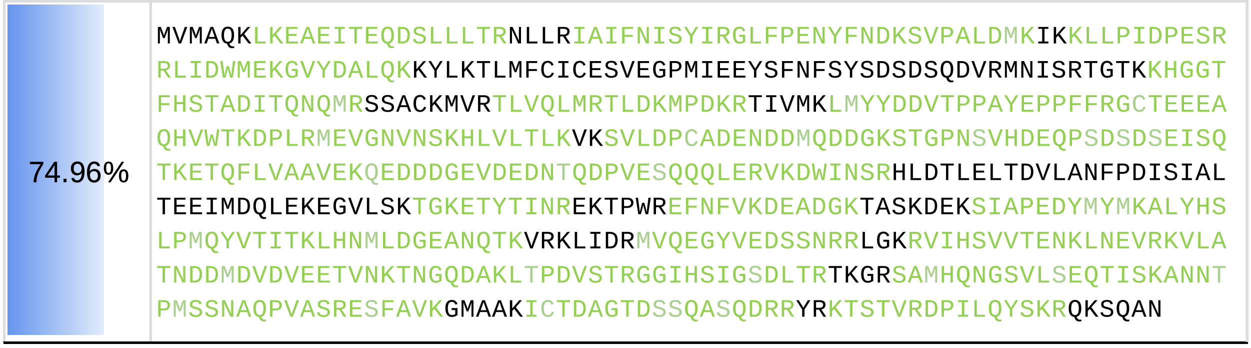

gi23506946 (BoASY1)

b

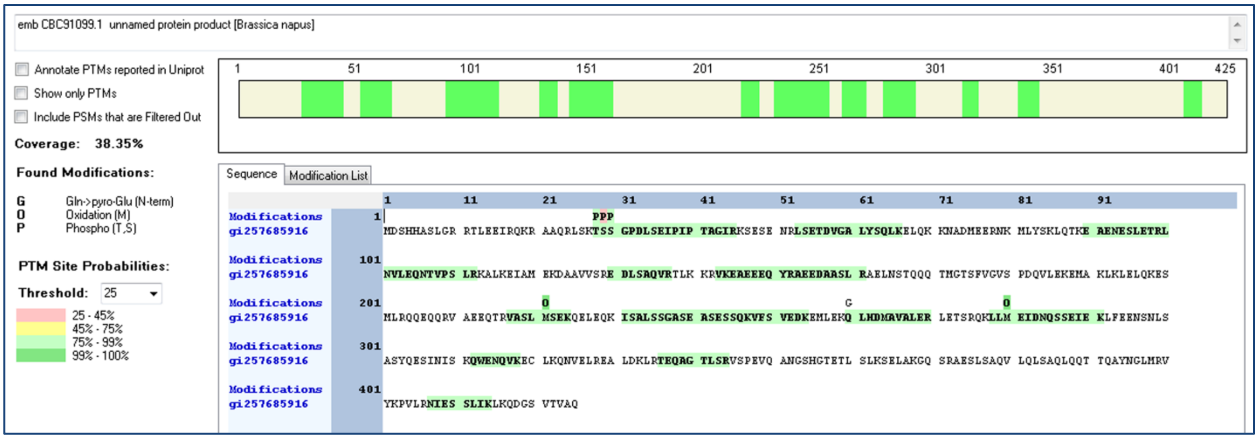

gi257685916 (SMC domain protein)

(T)SSGPDLSEIPIPTAGIR, T1 (Phospho):33.33 or T(S)SGPDLSEIPIPTAGIR, S2(Phospho):33.33 or TS(S)GPDLSEIPIPTAGIR, S3(Phospho):33.33

c

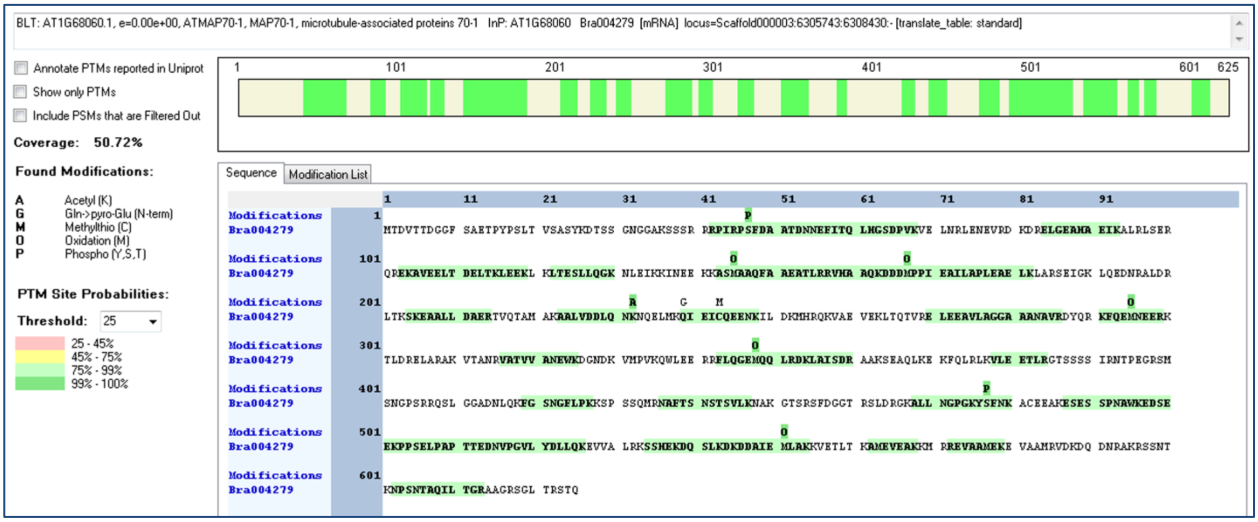

Bra004279 (MAP70-1 orthologue)

RPIRP(S)FDAATDNNEFITQLHGSDPVK, S6(Phospho):99.88 and ALLNGPGKY(S)FNK, S10(Phospho):99.32

Figure S2. Protein sequence coverage (**a**) BoASY1(gi23506946) (sequence coverage in green-type). (**b**) gi257685916, a SMC-domain protein and (**c**) Bra004279 (a MAP70-1 orthologue) showing phospho-modified peptides and their ptmRS best site probabilities (note that the precise position within the peptide could not be determined for the modification in gi257685916). Sequence coverage (highlighted green) and modifications were determined over all samples.
