# Supplementary material for: Affinity proteomics reveals extensive phosphorylation of the Brassica chromosome axis protein ASY1 and a network of associated proteins at prophase I of meiosis
Source: Plant J. 2017 Dec 2;93(1):17–33. doi: 10.1111/tpj.13752 (PMC5767750; doi:10.1111/tpj.13752)
Supplement: Supplementary file 3 — Figure S3. Mutant analysis of three meiotic candidates. [file TPJ-93-17-s003.pdf]

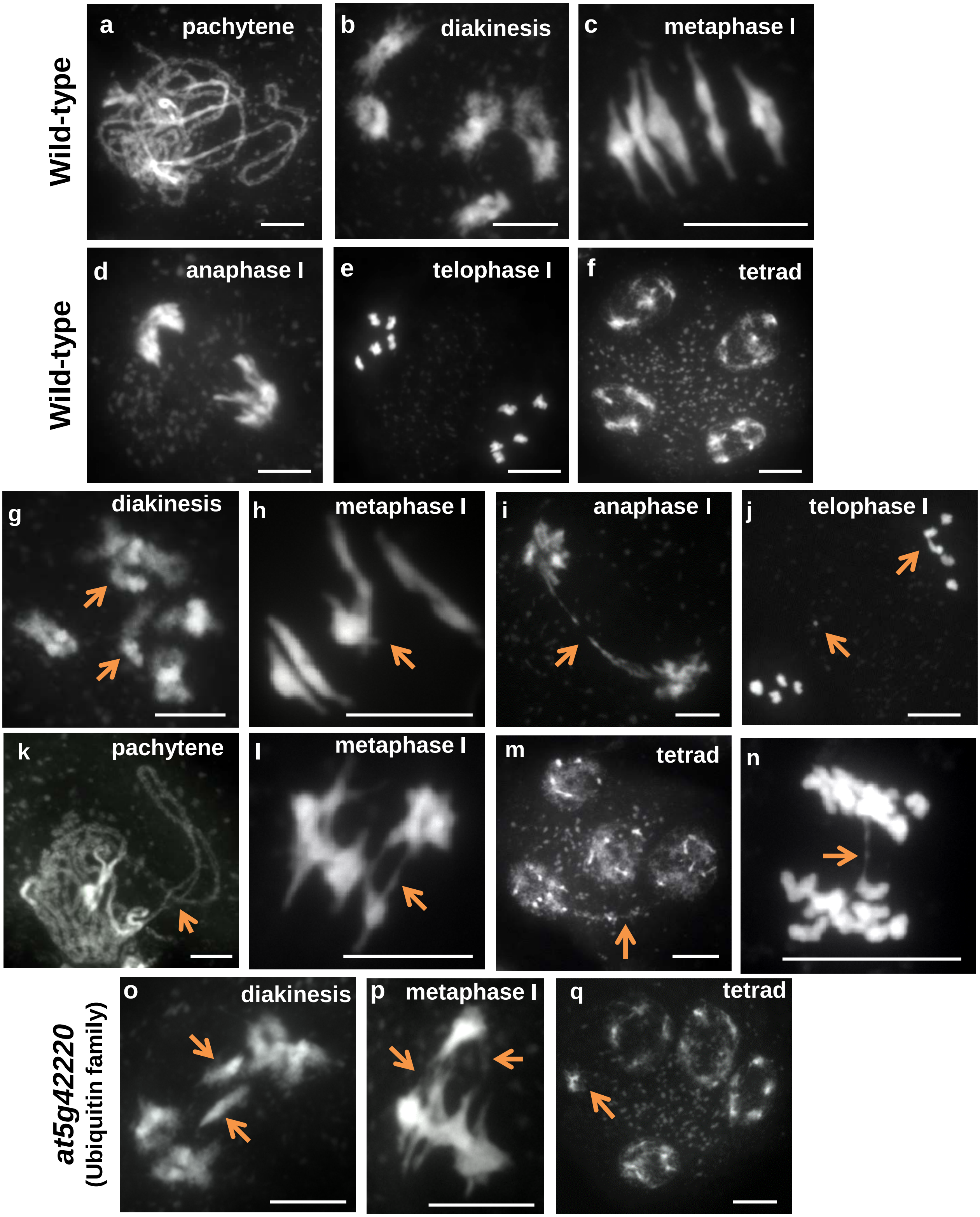

Figure S3. Mutant analysis of three meiotic candidates showing chromosome spreads of male meiocytes at various stages of meiosis. (**a-f**) WT. (**g-j**) *at5g46070* (SALK_016366) with: (**g**) univalents; (**h**) inter-bivalent connection, possibly an interlock; (**i**) chromosome bridge and (**j**) chromosome fragment and possible inter-chromosomal connection. (**k-n**) *at3g52140* (SALK_046271) with: (**k**) interlock; (**l**) inter-bivalent connections; (**m**) chromosome bridge and (**n**) mitotic chromosome bridge. (**o-q**) *at5g42220* (SALK_151742) with: (**o**) univalents; (**p**) inter-bivalent connections and (**q**) chromosome fragment. DNA is stained with DAPI. Abnormalities are indicated by arrows. Bar = 10 µm. Mutant identifiers are given in parentheses after gene identifiers. During the course of this study a role was proposed for At3g52140 in mediating inter-mitochondrial association (Zawily et al. 2014).
