# Supplementary material for: Affinity proteomics reveals extensive phosphorylation of the Brassica chromosome axis protein ASY1 and a network of associated proteins at prophase I of meiosis
Source: Plant J. 2017 Dec 2;93(1):17–33. doi: 10.1111/tpj.13752 (PMC5767750; doi:10.1111/tpj.13752)
Supplement: Supplementary file 4 — Figure S4. Mutant analysis of At5g59210. [file TPJ-93-17-s004.pdf]

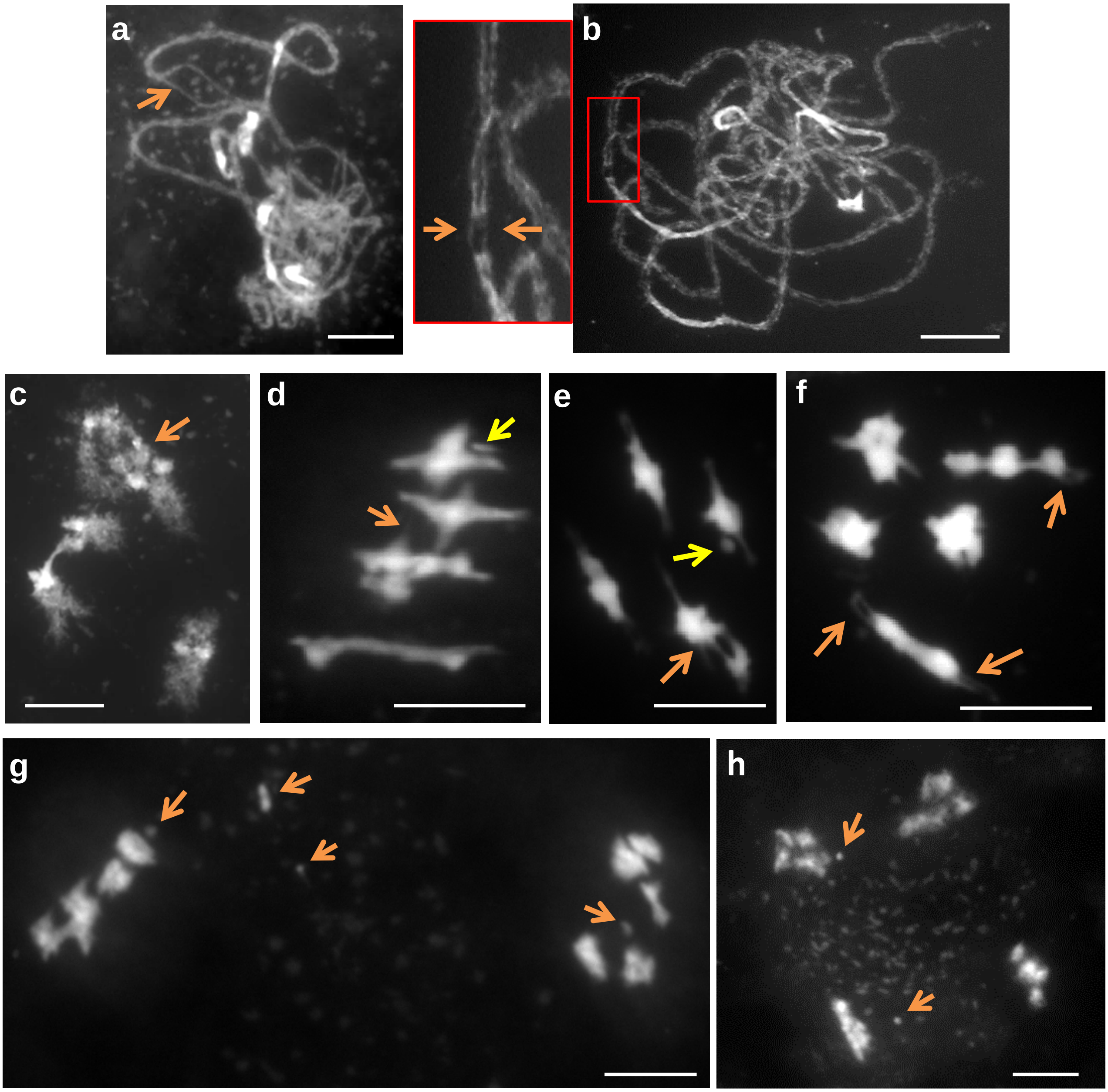


**Figure S4.** Mutant analysis of At5g59210.
Meiotic chromosome spreads of mutant line GABI_094G05 are stained with DAPI. (**a**) Pachytene with a small region of asynapsis. (**b**) Pachytene with a chromosomal region where both homologues have a gap in one sister chromatid, possibly due to incomplete repair of a recombination intermediate. See also zoomed inset. (**c**) Diakinesis with interlocked bivalents. (**d**, **e**) Metaphase I with chromosome fragment (yellow arrows) and inter-bivalent connections (orange arrows). (**f**) Metaphase I showing separation of sister chromatids close to centromeres. (**g**) Telophase I and (**h**) Telophase II with chromosome fragments. In each case, abnormalities are indicated by arrows. Bar = 10 µm.
