# Supplementary material for: Affinity proteomics reveals extensive phosphorylation of the Brassica chromosome axis protein ASY1 and a network of associated proteins at prophase I of meiosis
Source: Plant J. 2017 Dec 2;93(1):17–33. doi: 10.1111/tpj.13752 (PMC5767750; doi:10.1111/tpj.13752)
Supplement: Supplementary file 5 — Figure S5. Mutant analysis of meiotic candidate MCM2. [file TPJ-93-17-s005.pdf]

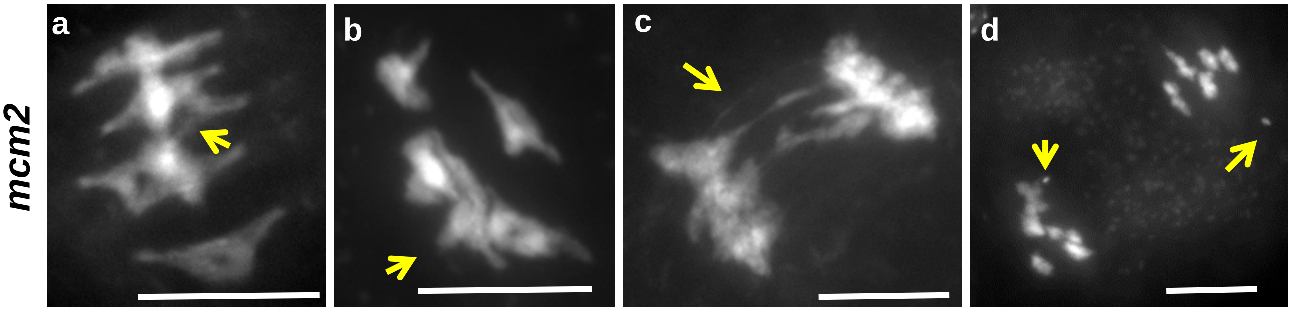

Figure S5. Mutant analysis of meiotic candidate MCM2 showing chromosome spreads of SALK_023429 at the first meiotic division. (a,b) Metaphase I with inter-bivalent connections. (c) Chromosome bridges at anaphase I. (d) Chromosome fragmentation after the first division. DNA is stained with DAPI. Features of interest are arrowed. Bar = 10 µm. For WT comparison refer to Supporting Figure S3.
