# Supplementary material for: Affinity proteomics reveals extensive phosphorylation of the Brassica chromosome axis protein ASY1 and a network of associated proteins at prophase I of meiosis
Source: Plant J. 2017 Dec 2;93(1):17–33. doi: 10.1111/tpj.13752 (PMC5767750; doi:10.1111/tpj.13752)
Supplement: Supplementary file 6 — Figure S6. Alignment of ASY4 (At2g33793) with ASY3 and mapping of T‐DNA insertion SAIL_886_D04 in asy4. [file TPJ-93-17-s006.pdf]

a

```
At2g33793           1 MSSTRRGTKRTRPEPPQSLKKPTPK-------AKLPDELDVDVSSDFKGI     43
                      .:|..:|..|....||..|.|...|       :::.::.|..:.......
ASY3              584 RNSNLKGIGRVVLSPPSPLSKGIDKTDSFQHCSEMDEDEDEGLGRAVALF    633

At2g33793          44 MSALQQFREKAHEDGRKKKEESISSVSTEVKSKIDELKSKLEKERQNFSK     93
                      ..|||.|..|......||..|.|:|||.|:..:::.:||.:..|....|.
ASY3              634 AMALQNFERKLKSAAEKKSSEIIASVSEEIHLELENIKSHIITEAGKTSN    683

At2g33793          94 ALSKSSKECENILKDEAAKFEELHKKFVKDKADHLQGLKDTISKFEEDKE    143
                      ......|..|..|:::..|...:|:||..|.:.||:..|.||.:.|.::.
ASY3              684 LAKTKRKHAETRLQEQEEKMRMIHEKFKDDVSHHLEDFKSTIEELEANQS    733

At2g33793         144 RLYMRYEQLRKKEKTMITEQEKFCTEKL---AQLEESLKKKKRGDKTFSI    190
                      .|....::.|...:.:|...|.....||   .:..:|:.|..|| |...
ASY3              734 ELKGSIKKQRTSHQKLIAHFEGGIETKLDDATKRIDSVNKSARG-KMLQ-    781

At2g33793         191 LRKTLGSFLENEASDEEFPPDE    212
                      |:..:...|.::
ASY3              782 LKMIVAECLRDD----------    793
```

b

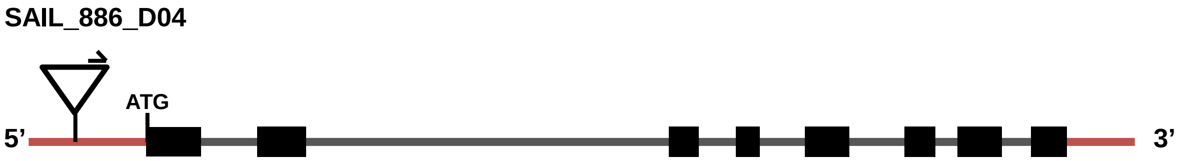

c

```
cagtgactttttgttacccacaatttgaagaattatatgcatagaataatttttatttttataatagtaattatg
ttagccataacttaaagaagaaaaaaaaatcaaaaaagaaaacgaaaatagtgagagtacgtgatcagctacgac
ggcatccacaatacgtaagtgggctatatattattgtgaagtgtgaagtcttctaacaaacaaacagaatttcaa
aattcaaattcaaatccaaatcacacgagcgagagagagcagataatcgccactcgccagagctattgatttcgc
ttcatcgaatctcacactctctaagtttcgtctgcaATGTCGTCTACCAGAAGAGGCACTAAGAGAACTCGGCCA
GAGCCGCCGCAATCGCTCAAGAAGCCAACACCGAAAGCAAAACTCCCGGACGAATTGGATGTCGATGTTTCTAGg
tccgttctcgtgtttcttttgcttcgtcttcttctttttcttcgtttcttctgtttttttgctgaaatgtgact
cgcgattgtgtttccgtgttgatcgtgtagCGATTTCAAAGGAATCATGTCGGCGTTGCAGCAATTCAGAGAGAA
AGCGCACGAAGATGGCCGTAAGAAGAAGGAAGAAAGCATTTCCAGgtcatatatctctccgttacatgtttctct
ctgattaggttatgtaaacttcgatttcatcattcactatgcagttatcgagatattcatgtcgatcgctcatgt
g.....
```

Figure S6. Alignment of ASY4 **(**At2g33793) with ASY3 and mapping of T-DNA insertion SAIL_886_D04 in *asy4*. (**a**) The full-length protein product of At2g33793 (ASY4) and the C-terminal region of ASY3 (amino acid residues 584-793) were aligned using EMBOSS Needle. Yellow highlight indicates predicted coiled-coil region in ASY3. (**b**) Position of SAIL_886_D04 in *asy4*, 160 bp upstream from translation start site. Exons are indicated by black boxes, introns by grey lines and UTR by brown lines. (**c**) Genomic sequence around insertion site. T-DNA is located between the t and a highlighted in pink. Intergenic regions are in black type, 5' UTR in red, exons in orange and introns in purple.
