# Supplementary material for: Affinity proteomics reveals extensive phosphorylation of the Brassica chromosome axis protein ASY1 and a network of associated proteins at prophase I of meiosis
Source: Plant J. 2017 Dec 2;93(1):17–33. doi: 10.1111/tpj.13752 (PMC5767750; doi:10.1111/tpj.13752)
Supplement: Supplementary file 12 — Appendix S2. Supporting experimental procedures. [file TPJ-93-17-s012.docx]

**Supporting Experimental Procedures**

**Primer sequences used in this study**

**T-DNA mutant line genotyping**

| **Gene** | **T-DNA line** | **Allele** | **Primer pairs** |
| --- | --- | --- | --- |
| At5g46070 | SALK_016366 | WT | 5'-CATGTCTGAATATTGATCATGCATTTTCACG-3' |
|  |  |  | 5'-GACGGAGAAGCAGAATCAGCC-3' |
|  |  | Mutant | 5'-CATGTCTGAATATTGATCATGCATTTTCACG-3' |
|  |  |  | 5'-GTGCTTTACGGCACCTCGAC-3' |
| At3g52140 | SALK_046271 | WT | 5'-CTTTAATTAGGATATTACAGTTCTTTGGAGC-3' |
|  |  |  | 5'-GGAGTGCTTAATTACGGGCCG-3' |
|  |  | Mutant | 5'-CTTTAATTAGGATATTACAGTTCTTTGGAGC-3' |
|  |  |  | 5'-GTGCTTTACGGCACCTCGAC-3' |
| At5g42220 | SALK_151742 | WT | 5'-CTTTCAGTATAGTTGATAACTTGACATCG-3' |
|  |  |  | 5'-GTAACAACACCTGATTCTCAACAATCC-3' |
|  |  | Mutant | 5'-GTAACAACACCTGATTCTCAACAATCC-3' |
|  |  |  | 5'-GTGCTTTACGGCACCTCGAC-3' |
| At5g59210 | GABI_094G05 | WT | 5'-GAGTTTCCCCAGACCAACTACC-3' |
|  |  |  | 5'-CAACAGCCATATCATGCAACTGC-3' |
|  |  | Mutant | 5'-GAGTTTCCCCAGACCAACTACC-3' |
|  |  |  | 5'-ATATTGACCATCATACTCATTGC-3' |
| At5g18620 | SALK_085156 | WT | 5'-GATGTAGAGAAAGGCTTACCTCC-3' |
|  |  |  | 5'-CCTTACCAGTCACTATCATAGAGG-3' |
|  |  | Mutant | 5'-GATGTAGAGAAAGGCTTACCTCC-3' |
|  |  |  | 5'-GTGCTTTACGGCACCTCGAC-3' |
| At1g44900 | SALK_023429 | WT | 5'-CTAAACATCATGTGGCTTAATCTCC-3' |
|  |  |  | 5'-GATCCAACTCGAAGTGAGCTTTGG-3' |
|  |  | Mutant | 5'-CTAAACATCATGTGGCTTAATCTCC-3' |
|  |  |  | 5'-GTGCTTTACGGCACCTCGAC-3' |
| At2g33793 | SAIL_886_D04 | WT | 5'-CGCATTTTTAAGCCATGAGGCCC-3' |
|  |  |  | 5'-CACAATCGCGAGTCACATTTCAGC-3' |
|  |  | Mutant | 5'-CACAATCGCGAGTCACATTTCAGC-3' |
|  |  |  | 5'-GCTTCCTATTATATCTTCCCAAATTACC-3' |

***icu2-1* genotyping by tetra-primer ARMS-PCR (Ye et al. 2001)**

5'-CTTAGCACAACAGATTCATCCGGTGGTGTATC-3'

5'-GGACTTGTTCCTTGAATCTCTGCGCATAGTCA-3'

5'-CCTTTCTTCATCGCTTGTTGCGAATAAGAGGG-3'

5'-GGTTGCACTCAGAATGAGGCAACGTGG-3'

**AtZYP1B-C antibody production**

ZYP1B-C-F: CCGCTAGCAAGCTGCAAGAAGAATTAGATCTTCAAAG

ZYP1B-C-R: CCCTCGAGATCAAATGCATAGGGATCATCAGC

**Yeast 2-hybrid cloning**

| ASY1 ^1-596^ | pGBKT7 | 5'-AGGAGGACCTGCATATGGTGATGGCTCAGAAGCTGAA-3' |
| --- | --- | --- |
|  |  | 5'-GGATCCCCGGGAATTCAATTAGCTTGAGATTTCTGACGCTT-3' |
| ASY1 ^1-596^ | pGADT7 | 5'-CAGATTACGCTCATATGGTGATGGCTCAGAAGCTGAA-3' |
|  |  | 5'-CACCCGGGTGGAATTCAATTAGCTTGAGATTTCTGACGCTT-3' |
| ASY3 ^1-793^ | pGADT7 | 5'-CAGATTACGCTCATATGAGCGACTATAGAAGCTTC-3' |
|  |  | 5'-CACCCGGGTGGAATTTCAATCATCCCTCAAACATTCTG-3' |
| ASY3 ^623-793^ | pGADT7 | 5'-CAGATTACGCTCATGAAGGCTTGGGAAGGGCTGTT-3' |
|  |  | 5'-CACCCGGGTGGAATTTCAATCATCCCTCAAACATTCTG-3' |
| ASY4 ^1-212^ | pGBKT7 | 5'-AGGAGGACCTGCATATGTCGTCTACCAGAAGAGGCAC-3' |
|  |  | 5'-CAGGTCGACGGATCCTCACTCATCAGGTGGGAATTCC-3' |
| ASY4 ^1-212^ | pGADT7 | 5'-CAGATTACGCTCATATGTCGTCTACCAGAAGAGGCAC-3' |
|  |  | 5'-GAGCTCGATGGATCCTCACTCATCAGGTGGGAATTCC-3' |

**Co-immunoprecipitation analysis**

Anthers (or extruded meiocytes) from 200 prophase I buds were powdered in liquid nitrogen and proteins extracted in a non-denaturing IP buffer (20 mM Tris-HCl pH 7.5, 150 mM NaCl, 10% glycerol, 2 mM EDTA, 0.1% NP40) containing protease inhibitor cocktail (Roche, # 04693159001) and phosphatase inhibitor cocktail (ThermoFisher Scientific, # 78420). All subsequent steps were performed at 4°C or on ice unless otherwise stated. Cell debris was removed by centrifugation (2 X 14,000 g for 10 minutes). Extracts were treated with PVPP using end-over-end rotation for 10 minutes to remove polyphenols and pre-cleared for 30 minutes with non-specificIgG. Parallel co-IPs were carried out for 30 minutes using anti-AtASY1 antibody or control rabbit IgG (Sigma-Aldrich, # 15006) cross-linked to Affi-Prep Protein A beads (Bio-Rad, # 156-0006). Following extensive washing (3 X 5 minutes with 40 bead-volumes of ice-cold IP buffer, followed by 3 X 5 minutes with 20 bv of IP buffer minus NP40 and protease and phosphatase inhibitors), bound proteins were eluted with 100 mM glycine pH 2, reduced by dithiothreitol, alkylated with methyl methanethiosulfonate and digested in solution with 400 ng trypsin (Trypsin Gold, MS grade, Promega). The generated peptides were analysed by nano-reversed phase-HPLC coupled online to an LTQ-Orbitrap Velos mass spectrometer (Thermo Fisher Scientific, Bremen, Germany) equipped with a Proxeon nanospray source (Proxeon, Odense, Denmark). The nano HPLC system used was an UltiMate 3000 HPLC RSLC nano system (Thermo Fisher Scientific, Bremen, Germany). Peptides were loaded onto a trap column (Thermo Fisher Scientific, Bremen, Germany, PepMap C18, 5 mm × 300 μm ID, 5 μm particles, 100 Å pore size) at a flow rate of 25 μL min^-1^ using 0.1% TFA as mobile phase. After 10 min, the trap column was switched in line with the analytical column (Thermo Fisher Scientific, Bremen, Germany, PepMap C18, 500 mm × 75 μm ID, 3 μm, 100 Å). Peptides were eluted using a flow rate of 230 nl min^-1^, and a binary 2h gradient. The gradient starts with the mobile phases: 98% A (water/formic acid, 99.9/0.1, v/v) and 2% B (water/acetonitrile/formic acid, 19.92/80/0.08, v/v/v) increases to 35% B over the next 120 min, followed by a gradient in 5 min to 90% B, stays there for five min and decreases in 5 min back to the gradient 98% A and 2% B for equilibration at 30°C.

The LTQ Orbitrap Velos was operated in data-dependent mode, using a full scan in the Orbitrap (m/z range 350-2000, nominal resolution of 60 000, target value 1E6) followed by MS/MS scans of the 12 most abundant ions in the linear ion trap. MS/MS spectra (normalized collision energy 35%; activation value q 0.25; activation time 10 ms; isolation width 2, target value 1E4) were acquired and subsequent activation was performed on fragment ions through multistage activation. The neutral loss mass list was therefore set to -98, -49, and -32.6 m/z. Precursor ions selected for fragmentation (charge state 2 and higher) were put on a dynamic exclusion list for 60 s. Additionally, singly-charged parent ions were excluded from selection for MS/MS experiments and the monoisotopic precursor selection feature was enabled.

MS/MS spectra were interpreted using Mascot (version 2.1.0.81, Matrix Science, London, UK) in conjunction with Proteome Discoverer software (version 1.4.0.288, Thermo Fisher Scientific, MA, USA). Peptides and proteins were identified by searching against *B. rapa* sequences (Brassica_rapa.20100830.pep,_version 1.2, 41173 sequences, downloaded from brassicadb.org), combined with Brassica sequences from NCBI (http://www.ncbi.nlm.nih.gov, comprising 13230 sequences from Brassica rapa, napus and oleracea) as at 2010, supplemented with common contaminants. The following search parameters were used: a precursor mass tolerance of 5 ppm; a fragment ion mass tolerance of 0.5 Da; two missed cleavage sites for trypsin were permitted; and fixed modification of cysteine with methyl methanethiosulfonate was specified. Oxidation of methionine, phosphorylation of serine, threonine and tyrosine, pyroglutamate formation of glutamine at the peptide N-termini and acetylation on lysine were specified as a variable modification. The result was validated using the Percolator algorithm as integrated in Proteome Discoverer and the PSM and protein lists were filtered to 1% FDR (false discovery rate) each. Additionally, a Mascot Ions Score cut-off of 8 was defined as the minimum acceptance threshold for the each peptide spectrum match and a minimum of two unique peptides were required to positively identify a protein, resulting in an actual FDR of below 1%.

The localization of the sites of variable modifications within the peptides was performed with the tool ptmRS, integrated in Proteome Discoverer and based on phosphoRS (Taus et al., 2011).

The in-house-developed tool Peakjuggler was used for label-free quantification of peptides and proteins (publication in preparation; Andersen et al. 2017). Protein quantification is based on the average area of the three most abundant peptides. Statistical significance of differentially abundant proteins was determined using limma (Smyth 2004).

**Supporting References**

**Bies‐Etheve, N., Pontier, D., Lahmy, S., Picart, C., Vega, D., Cooke, R. and Lagrange, T.** (2009) RNA‐directed DNA methylation requires an AGO4‐interacting member of the SPT5 elongation factor family. *EMBO reports*, **10**, 649.

**Callis, J.** (2014) The Ubiquitination Machinery of the Ubiquitin System. *The Arabidopsis Book / American Society of Plant Biologists*, **12**, e0174.

[**Casamayor A**](https://www.ncbi.nlm.nih.gov/pubmed/?term=Casamayor%20A%5BAuthor%5D&cauthor=true&cauthor_uid=7948902)**,** [**Pérez-Callejón E**](https://www.ncbi.nlm.nih.gov/pubmed/?term=P%C3%A9rez-Callej%C3%B3n%20E%5BAuthor%5D&cauthor=true&cauthor_uid=7948902)**,** [**Pujol G**](https://www.ncbi.nlm.nih.gov/pubmed/?term=Pujol%20G%5BAuthor%5D&cauthor=true&cauthor_uid=7948902)**,** [**Ariño J**](https://www.ncbi.nlm.nih.gov/pubmed/?term=Ari%C3%B1o%20J%5BAuthor%5D&cauthor=true&cauthor_uid=7948902)**, and** [**Ferrer A**](https://www.ncbi.nlm.nih.gov/pubmed/?term=Ferrer%20A%5BAuthor%5D&cauthor=true&cauthor_uid=7948902)**.** (1994) Molecular characterization of a fourth isoform of the catalytic subunit of protein phosphatase 2A from Arabidopsis thaliana. *Plant Molecular Biology* **26**, 523-528

**Chen, M. and Thelen, J.J.** (2011) Plastid Uridine Salvage Activity Is Required for Photoassimilate Allocation and Partitioning in Arabidopsis. *The Plant Cell*, **23**, 2991-3006.

**Cheng, S.H., Willmann, M.R., Chen, H.C. and Sheen, J.** (2002) Calcium Signaling through Protein Kinases. The Arabidopsis Calcium-Dependent Protein Kinase Gene Family. *Plant Physiology*, **129**, 469-485.

**Cheng, Y., Dai, X. and Zhao, Y.** (2004) AtCAND1, A HEAT-Repeat Protein That Participates in Auxin Signaling in Arabidopsis. *Plant Physiology*, **135**, 1020-1026.

**Chuang, H.W., Zhang, W. and Gray, W.M.** (2004) Arabidopsis ETA2, an Apparent Ortholog of the Human Cullin-Interacting Protein CAND1, Is Required for Auxin Responses Mediated by the SCFTIR1 Ubiquitin Ligase. *The Plant Cell*, **16**, 1883-1897.

**Cui, X., Lu, F., Li, Y., Xue, Y., Kang, Y., Zhang, S., Qiu, Q., Cui, X., Zheng, S., Liu, B., Xu, X. and Cao, X.** (2013) Ubiquitin-Specific Proteases UBP12 and UBP13 Act in Circadian Clock and Photoperiodic Flowering Regulation in Arabidopsis. *Plant Physiology*, **162**, 897.

**El Zawily, A.M., Schwarzländer, M., Finkemeier, I., Johnston, I.G., Benamar, A., Cao, Y., Gissot, C., Meyer, A.J., Wilson, K., Datla, R., Macherel, D., Jones, N.S. and Logan, D.C.** (2014) FRIENDLY Regulates Mitochondrial Distribution, Fusion, and Quality Control in Arabidopsis. *Plant Physiology*, **166**, 808-828.

**Ewan, R., Pangestuti, R., Thornber, S., Craig, A., Carr, C., O’Donnell, L., Zhang, C. and Sadanandom, A.** (2011) Deubiquitinating enzymes AtUBP12 and AtUBP13 and their tobacco homologue NtUBP12 are negative regulators of plant immunity. *New Phytologist*, **191**, 92-106.

**Farmer, L.M., Book, A.J., Lee, K.H., Lin, Y.L., Fu, H. and Vierstra, R.D.** (2010) The RAD23 Family Provides an Essential Connection between the 26S Proteasome and Ubiquitylated Proteins in Arabidopsis. *The Plant Cell*, **22**, 124-142.

**Feng, S., Shen, Y., Sullivan, J.A., Rubio, V., Xiong, Y., Sun, T.-p. and Deng, X.W.** (2004) Arabidopsis CAND1, an Unmodified CUL1-Interacting Protein, Is Involved in Multiple Developmental Pathways Controlled by Ubiquitin/Proteasome-Mediated Protein Degradation. *The Plant Cell*, **16**, 1870-1882.

**Frankard, V., Vauterin, M. and Jacobs, M.** (1997) Molecular characterization of an Arabidopsis thaliana cDNA coding for a monofunctional aspartate kinase. *Plant Molecular Biology*, **34**, 233-242.

**Giegé, P., Heazlewood, J.L., Roessner-Tunali, U., Millar, A.H., Fernie, A.R., Leaver, C.J. and Sweetlove, L.J.** (2003) Enzymes of Glycolysis Are Functionally Associated with the Mitochondrion in Arabidopsis Cells. *The Plant Cell*, **15**, 2140-2151.

**Grasser, K.D., Grill, S., Duroux, M., Launholt, D., Thomsen, M.S., Nielsen, B.V., Nielsen, H.K. and Merkle, T.** (2004) HMGB6 from Arabidopsis thaliana Specifies a Novel Type of Plant Chromosomal HMGB Protein. *Biochemistry*, **43**, 1309-1314.

**Herr, A.J., Jensen, M.B., Dalmay, T. and Baulcombe, D.C.** (2005) RNA Polymerase IV Directs Silencing of Endogenous DNA. *Science*, **308**, 118.

**Hill, J.E. and Hemmingsen, S.M.** (2001) Arabidopsis thaliana type I and II chaperonins. *Cell Stress & Chaperones*, **6**, 190-200.

**Hill, K., Wang, H. and Perry, S.E.** (2008) A transcriptional repression motif in the MADS factor AGL15 is involved in recruitment of histone deacetylase complex components. *The Plant Journal*, **53**, 172-185.

**Himelblau, E., Mira, H., Lin, S.J., Cizewski Culotta, V., Peñarrubia, L. and Amasino, R.M.** (1998) Identification of a Functional Homolog of the Yeast Copper Homeostasis Gene ATX1 from Arabidopsis. *Plant Physiology*, **117**, 1227-1234.

**Kanno, T., Huettel, B., Mette, M.F., Aufsatz, W., Jaligot, E., Daxinger, L., Kreil, D.P., Matzke, M. and Matzke, A.J.M.** (2005) Atypical RNA polymerase subunits required for RNA-directed DNA methylation. *Nat Genet*, **37**, 761-765.

**Kopczak, S.D., Haas, N.A., Hussey, P.J., Silflow, C.D. and Snustad, D.P.** (1992) The small genome of Arabidopsis contains at least six expressed alpha-tubulin genes. *The Plant Cell*, **4**, 539-547.

**Krishna, P. and Gloor, G.** (2001) The Hsp90 family of proteins in Arabidopsis thaliana. *Cell Stress & Chaperones*, **6**, 238-246.

**Krupnova, T., Sasabe, M., Ghebreghiorghis, L., Gruber, C.W., Hamada, T., Dehmel, V., Strompen, G., Stierhof, Y.-D., Lukowitz, W., Kemmerling, B., Machida, Y., Hashimoto, T., Mayer, U. and Jürgens, G.** (2009) Microtubule-Associated Kinase-like Protein RUNKEL Needed for Cell Plate Expansion in Arabidopsis Cytokinesis. *Current Biology*, **19**, 518-523.

**Launholt, D., Merkle, T., Houben, A., Schulz, A. and Grasser, K.D.** (2006) Arabidopsis Chromatin-Associated HMGA and HMGB Use Different Nuclear Targeting Signals and Display Highly Dynamic Localization within the Nucleus. *The Plant Cell*, **18**, 2904-2918.

**Li, B. and Kliebenstein, D.J.** (2014) The AT-hook motif-encoding gene METABOLIC NETWORK MODULATOR 1 underlies natural variation in Arabidopsis primary metabolism. *Frontiers in Plant Science*, **5**.

**Lim, G.H., Zhang, X., Chung, M.S., Lee, D.J., Woo, Y.M., Cheong, H.S. and Kim, C.S.** (2010) A putative novel transcription factor, AtSKIP, is involved in abscisic acid signalling and confers salt and osmotic tolerance in Arabidopsis. *New Phytologist*, **185**, 103-113.

**Mainguet, S.E., Gakière, B., Majira, A., Pelletier, S., Bringel, F., Guérard, F., Caboche, M., Berthomé, R. and Renou, J.P.** (2009) Uracil salvage is necessary for early Arabidopsis development. *The Plant Journal*, **60**, 280-291.

**Mantegazza, O., Gregis, V., Mendes, M.A., Morandini, P., Alves-Ferreira, M., Patreze, C.M., Nardeli, S.M., Kater, M.M. and Colombo, L.** (2014) Analysis of the arabidopsis REM gene family predicts functions during flower development. *Annals of Botany*, **114**, 1507-1515.

**Marks, M.D., West, J. and Weeks, D.P.** (1987) The relatively large beta-tubulin gene family of Arabidopsis contains a member with an unusual transcribed 5′ noncoding sequence. *Plant Molecular Biology*, **10**, 91-104.

**Milioni, D. and Hatzopoulos, P.** (1997) Genomic organization of hsp90 gene family in Arabidopsis. *Plant Molecular Biology*, **35**, 955-961.

**Okada, T., Endo, M., Singh, M.B. and Bhalla, P.L.** (2005) Analysis of the histone H3 gene family in Arabidopsis and identification of the male-gamete-specific variant AtMGH3. *The Plant Journal*, **44**, 557-568.

**Onodera, Y., Haag, J.R., Ream, T., Nunes, P.C., Pontes, O. and Pikaard, C.S.** (2005) Plant Nuclear RNA Polymerase IV Mediates siRNA and DNA Methylation-Dependent Heterochromatin Formation. *Cell*, **120**, 613-622.

**Parsley, K. and Hibberd, J.M.** (2006) The Arabidopsis PPDK gene is transcribed from two promoters to produce differentially expressed transcripts responsible for cytosolic and plastidic proteins. *Plant Molecular Biology*, **62**, 339-349.

[**Pérez-Callejón E**](https://www.ncbi.nlm.nih.gov/pubmed/?term=P%C3%A9rez-Callej%C3%B3n%20E%5BAuthor%5D&cauthor=true&cauthor_uid=8292782)**,** [**Casamayor A**](https://www.ncbi.nlm.nih.gov/pubmed/?term=Casamayor%20A%5BAuthor%5D&cauthor=true&cauthor_uid=8292782)**,** [**Pujol G**](https://www.ncbi.nlm.nih.gov/pubmed/?term=Pujol%20G%5BAuthor%5D&cauthor=true&cauthor_uid=8292782)**,** [**Clua E**](https://www.ncbi.nlm.nih.gov/pubmed/?term=Clua%20E%5BAuthor%5D&cauthor=true&cauthor_uid=8292782)**,** [**Ferrer A**](https://www.ncbi.nlm.nih.gov/pubmed/?term=Ferrer%20A%5BAuthor%5D&cauthor=true&cauthor_uid=8292782)**,** [**Ariño J**](https://www.ncbi.nlm.nih.gov/pubmed/?term=Ari%C3%B1o%20J%5BAuthor%5D&cauthor=true&cauthor_uid=8292782)**.**(1993) Identification and molecular cloning of two homologues of protein phosphatase X from Arabidopsis thaliana. *Plant Molecular Biology* **23**, 1177-1185

**Pontier, D., Yahubyan, G., Vega, D., Bulski, A., Saez-Vasquez, J., Hakimi, M.A., Lerbs-Mache, S., Colot, V. and Lagrange, T.** (2005) Reinforcement of silencing at transposons and highly repeated sequences requires the concerted action of two distinct RNA polymerases IV in Arabidopsis. *Genes & Development*, **19**, 2030-2040.

**Ream, T.S., Haag, J.R., Wierzbicki, A.T., Nicora, C.D., Norbeck, A.D., Zhu, J.-K., Hagen, G., Guilfoyle, T.J., Paša-Tolić, L. and Pikaard, C.S.** (2009) Subunit Compositions of the RNA-Silencing Enzymes Pol IV and Pol V Reveal Their Origins as Specialized Forms of RNA Polymerase II. *Molecular Cell*, **33**, 192-203.

**Riechmann, J.L., Heard, J., Martin, G., Reuber, L., Jiang, C.Z., Keddie, J., Adam, L., Pineda, O., Ratcliffe, O.J., Samaha, R.R., Creelman, R., Pilgrim, M., Broun, P., Zhang, J.Z., Ghandehari, D., Sherman, B.K. and L. Yu, G.** (2000). Arabidopsis Transcription Factors: Genome-Wide Comparative Analysis Among Eukaryotes. *Science*, **290**, 2105.

[**Rudrabhatla P**](https://www.ncbi.nlm.nih.gov/pubmed/?term=Rudrabhatla%20P%5BAuthor%5D&cauthor=true&cauthor_uid=16429265)**.,** [**Reddy MM**](https://www.ncbi.nlm.nih.gov/pubmed/?term=Reddy%20MM%5BAuthor%5D&cauthor=true&cauthor_uid=16429265)**., and** [**Rajasekharan R**](https://www.ncbi.nlm.nih.gov/pubmed/?term=Rajasekharan%20R%5BAuthor%5D&cauthor=true&cauthor_uid=16429265)**.** (2006) Genome-wide analysis and experimentation of plant serine/ threonine/tyrosine-specific protein kinases. *Plant Molecular Biology*, **60**, 293-319.

**Salie, M.J., Zhang, N., Lancikova, V., Xu, D. and Thelen, J.J.** (2016) A Family of Negative Regulators Targets the Committed Step of de Novo Fatty Acid Biosynthesis. *The Plant Cell*, **28**, 2312-2325.

**Schubert, V., Lermontova, I. and Schubert, I.** (2013) The Arabidopsis CAP-D proteins are required for correct chromatin organisation, growth and fertility. *Chromosoma*, **122**, 517-533.

**Shikata, M., Matsuda, Y., Ando, K., Nishii, A., Takemura, M., Yokota, A. and Kohchi, T.** (2004) Characterization of Arabidopsis ZIM, a member of a novel plant‐specific GATA factor gene family. *Journal of Experimental Botany*, **55**, 631-639.

**Slabas, A.R., Fordham-Skelton, A.P., Fletcher, D., Martinez-Rivas, J.M., Swinhoe, R., Croy, R.R.D. and Evans, I.M.** (1994) Characterisation of cDNA and genomic clones encoding homologues of the 65 kDa regulatory subunit of protein phosphatase 2A in Arabidopsis thaliana. *Plant Molecular Biology*, **26**, 1125-1138.

**Stemmer, C., Ritt, C., Igloi, G.L., Grimm, R. and Crasser, K.D.** (1997) Variability in Arabidopsis Thaliana Chromosomal High-Mobility-Group-1-Like Proteins. *European Journal of Biochemistry*, **250**, 646-652.

**Storozhenko, S., De Pauw, P., Kushnir, S., Van Montagu, M. and Inzé, D.** (1996) Identification of an Arabidopsis thaliana cDNA encoding a HSP70-related protein belonging to the HSP110/SSE1 subfamily. *FEBS Letters*, **390**, 113-118.

**Sung, D.Y., Vierling, E. and Guy, C.L.** (2001) Comprehensive Expression Profile Analysis of the Arabidopsis Hsp70 Gene Family. *Plant Physiology*, **126**, 789-800.

**Tang, G., Zhu-Shimoni, J.X., Amir, R., Ben-Tzvi Zchori, I. and Galili, G.** (1997) Cloning and expression of an Arabidopsis thaliana cDNA encoding a monofunctional aspartate kinase homologous to the lysine-sensitive enzyme of Escherichia coli. *Plant Molecular Biology*, **34**, 287-294.

**Taus, T., Köcher, T., Pichler, P., Paschke, C., Schmidt, A., Henrich, C. and Mechtler, K.** (2011) Universal and Confident Phosphorylation Site Localization Using phosphoRS. *Journal of Proteome Research*, **10**, 5354-5362.

**Turner, W.L., Knowles, V.L. and Plaxton, W.C.** (2005) Cytosolic pyruvate kinase: subunit composition, activity, and amount in developing castor and soybean seeds, and biochemical characterization of the purified castor seed enzyme. *Planta*, **222**, 1051-1062.

**Xie, M., Ren, G., Costa-Nunes, P., Pontes, O. and Yu, B.** (2012) A subgroup of SGS3-like proteins act redundantly in RNA-directed DNA methylation. *Nucleic Acids Research*, **40**, 4422-4431.

**Xie, S. and Lam, E.** (1994) Abundance of nuclear DNA topoisomerase II is correlated with proliferation in Arabidopsis thaliana. *Nucleic Acids Research*, **22**, 5729-5736.

**Xu, J., Yang, C., Yuan, Z., Zhang, D., Gondwe, M.Y., Ding, Z., Liang, W., Zhang, D. and Wilson, Z.A.** (2010) The ABORTED MICROSPORES Regulatory Network Is Required for Postmeiotic Male Reproductive Development in Arabidopsis thaliana. *The Plant Cell*, **22**, 91-107.

**Yan, N., Doelling, J.H., Falbel, T.G., Durski, A.M. and Vierstra, R.D.** (2000) The Ubiquitin-Specific Protease Family from Arabidopsis. UBP1 and 2 Are Required for the Resistance to the Amino Acid Analog Canavanine. *Plant Physiology*, **124**, 1828.

**Yang, J., Tian, L., Sun, M.-X., Huang, X.-Y., Zhu, J., Guan, Y.-F., Jia, Q.-S. and Yang, Z.-N.** (2013) AUXIN RESPONSE FACTOR17 Is Essential for Pollen Wall Pattern Formation in Arabidopsis. *Plant Physiology*, **162**, 720.

**Yu, D., Fan, B., MacFarlane, S.A. and Chen, Z.** (2003) Analysis of the Involvement of an Inducible Arabidopsis RNA-Dependent RNA Polymerase in Antiviral Defense. *Molecular Plant-Microbe Interactions*, **16**, 206-216.

**Zheng, B., Wang, Z., Li, S., Yu, B., Liu, J. Y. and Chen, X.** (2009) Intergenic transcription by RNA Polymerase II coordinates Pol IV and Pol V in siRNA-directed transcriptional gene silencing in Arabidopsis. *Genes & Development*, **23**, 2850-2860.

**Zhou, R., Kroczynska, B., Hayman, G.T. and Miernyk, J.A.** (1995) AtJ2, an arabidopsis homolog of Escherichia coli dnaJ. *Plant Physiology*, **108**, 821-822.

**Zilberman, D., Cao, X. and Jacobsen, S.E.** (2003) ARGONAUTE4 Control of Locus-Specific siRNA Accumulation and DNA and Histone Methylation. *Science*, **299**, 716.

**Zilberman, D., Cao, X., Johansen, L.K., Xie, Z., Carrington, J.C. and Jacobsen, S.E.** (2004) Role of Arabidopsis ARGONAUTE4 in RNA-Directed DNA Methylation Triggered by Inverted Repeats. *Current Biology*, **14**,
