# Supplementary material for: Affinity proteomics reveals extensive phosphorylation of the Brassica chromosome axis protein ASY1 and a network of associated proteins at prophase I of meiosis
Source: Plant J. 2017 Dec 2;93(1):17–33. doi: 10.1111/tpj.13752 (PMC5767750; doi:10.1111/tpj.13752)
Supplement: Supplementary file 13 [file TPJ-93-17-s013.docx]

**Supporting Information Legends**

**Figure S1.** Targeting BoASY1 using an anti-AtASY1 antibody. (a) Full-length alignment of AtASY1 and BoASY1 using EMBOSS Needle (www.ebi.ac.uk). (b) Immunolocalisation of ASY1 (green) at leptotene in *B. oleracea* meiocyte. DNA is stained with DAPI (blue). (c) Immunoblot of ASY1 in *B. oleracea* meiotic tissues (loading is equivalent to 10 anthers per lane).

**Figure S2. Figure S2. Protein sequence coverage** (**a**) BoASY1(gi23506946) (sequence coverage in green-type). (**b**) gi257685916, a SMC-domain protein and (**c**) Bra004279 (a MAP70-1 orthologue) showing phospho-modified peptides and their ptmRS best site probabilities (note that the precise position within the peptide could not be determined for the modification in gi257685916). Sequence coverage (highlighted green) and modifications were determined over all samples.

**Figure S3.** Mutant analysis of three meiotic candidates showing chromosome spreads of male meiocytes at various stages of meiosis. (a-f) WT. (g-j) *at5g46070* (SALK_016366) with: (g) univalents; (h) inter-bivalent connection, possibly an interlock; (i) chromosome bridge and (j) chromosome fragment and possible inter-chromosomal connection. (k-n) a*t3g52140* (SALK_046271) with: (k) interlock; (l) inter-bivalent connections; (m) chromosome bridge and (n) mitotic chromosome bridge. (o-q) *at5g42220* (SALK_151742) with: (o) univalents; (p) inter-bivalent connections and (q) chromosome fragment. DNA is stained with DAPI. Abnormalities are indicated by arrows. Bar = 10 µm. Mutant identifiers are given in parentheses after gene identifiers. During the course of this study a role was proposed for At3g52140 in mediating inter-mitochondrial association (Zawily et al. 2014).

**Figure S4.** Mutant analysis of At5g59210. Meiotic chromosome spreads of mutant line GABI_094G05 are stained with DAPI. (**a**) Pachytene with a small region of asynapsis. (**b**) Pachytene with a chromosomal region where both homologues have a gap in one sister chromatid, possibly due to incomplete repair of a recombination intermediate. See also zoomed inset. (**c**) Diakinesis with interlocked bivalents. (**d**, **e**) Metaphase I with chromosome fragment (yellow arrows) and inter-bivalent connections (orange arrows). (**f**) Metaphase I showing separation of sister chromatids close to centromeres. (**g**) Telophase I and (**h**) Telophase II with chromosome fragments. In each case, abnormalities are indicated by arrows. Bar = 10 µm.

**Figure S5.** Mutant analysis of meiotic candidate MCM2 showing chromosome spreads of SALK_023429 at the first meiotic division. (a,b) Metaphase I with inter-bivalent connections. (c) Chromosome bridges at anaphase I. (d) Chromosome fragmentation after the first division. DNA is stained with DAPI. Features of interest are arrowed. Bar = 10 µm. For WT comparison refer to Figure S3.

**Figure S6.** Alignment of ASY4 (At2g33793) with ASY3 and mapping of T-DNA insertion SAIL_886_D04 in *asy4*. (a) The full-length protein product of At2g33793 (ASY4) and the C-terminal region of ASY3 (amino acid residues 584-793) were aligned using EMBOSS Needle. Yellow highlight indicates predicted coiled-coil region in ASY3. (b) Position of SAIL_886_D04 in *asy4*, 160 bp upstream from translation start site. Exons are indicated by black boxes, introns by grey lines and UTR by brown lines. (c) Genomic sequence around insertion site. T-DNA is located between the t and a highlighted in pink. Intergenic regions are in black type, 5’ UTR in red, exons in orange and introns in purple.

**Table S1.** ASY1 sample-specific Brassica proteins with their putative Arabidopsis orthologues. Proteins showing a fold-change (FC) of ≥5 in sample relative to control and p<0.01 in at least one dataset were considered quantitatively significant and are marked in green. Proteins which were identified with at least 2 peptides in the sample but with none in the control within the whole dataset (marked 'TRUE' in ≥ 2 vs 0 Peptides column), but were not quantitatively significant, or were identified exclusively in those datasets for which Limma analysis was not possible, were accepted as a less reliable group and are marked in orange. For these proteins, the relevant number of peptides are shown in additional columns. The quantitatively significant group of proteins are ranked as follows: No. Datasets with significant FC, then 'TRUE' in ≥ 2 vs 0 Peptides column, then Max. FC. The qualitatively accepted group are ranked according to number of peptides in sample. For ease of interpretation, orthologues specifying identical Arabidopsis IDs are grouped together and a column showing STRING names is included for cross-referencing with the PPI network in Data_S1.

**Table S2.** Gene Ontology enrichment analysis of ASY1 sample-specific proteins. GO analysis of putative A. thaliana orthologues against the A. thaliana genome was carried out using PANTHER accessed through the GO consortium web-site (http://geneontology.org). (a) Biological process. (b) Molecular Function. (c) Cellular component.

**Table S3.** Functional grouping of ASY1 sample-specific proteins. Where possible, proteins were grouped using KEGG pathway analysis of putative Arabidopsis orthologues and the relevant literature as appropriate (KEGG, http://www.genome.jp/kegg/pathway.html).

**Table S4.** Summary of analysis of meiotic candidates.

**Appendix S1.** Protein-Protein Interaction (PPI) network of ASY1 sample-specific proteins in Cytoscape format. PPI data were from STRING DB v10.5. Nodes represent proteins, edges represent interactions.
